# *OsPK2* encodes a plastidic pyruvate kinase involved in rice endosperm starch synthesis, compound granule formation and grain filling

**DOI:** 10.1111/pbi.12923

**Published:** 2018-04-17

**Authors:** Yicong Cai, Sanfeng Li, Guiai Jiao, Zhonghua Sheng, Yawen Wu, Gaoneng Shao, Lihong Xie, Cheng Peng, Junfeng Xu, Shaoqing Tang, Xiangjin Wei, Peisong Hu

**Affiliations:** ^1^ State Key Laboratory of Rice Biology China National Rice Research Institute Hangzhou China; ^2^ State Key Laboratory Breeding Base for Zhejiang Sustainable Pest and Disease Control Institute of Quality and Standard for Agro‐products Zhejiang Academy of Agricultural Sciences Hangzhou China

**Keywords:** grain filling, amyloplast development, pyruvate kinase, starch biosynthesis, rice

## Abstract

Starch is the main form of energy storage in higher plants. Although several enzymes and regulators of starch biosynthesis have been defined, the complete molecular machinery remains largely unknown. Screening for irregularities in endosperm formation in rice represents valuable prospect for studying starch synthesis pathway. Here, we identified a novel rice white‐core endosperm and defective grain filling mutant, *ospk2*, which displays significantly lower grain weight, decreased starch content and alteration of starch physicochemical properties when compared to wild‐type grains. The normal starch compound granules were drastically reduced and more single granules filled the endosperm cells of *ospk2*. Meanwhile, the germination rate of *ospk2* seeds after 1‐year storage was observably reduced compared with wild‐type. Map‐based cloning of *OsPK2* indicated that it encodes a pyruvate kinase (PK, ATP: pyruvate 2‐O‐phosphotransferase, EC 2.7.1.40), which catalyses an irreversible step of glycolysis. *OsPK2* has a constitutive expression in rice and its protein localizes in chloroplasts. Enzyme assay showed that the protein product from expressed *OsPK2* and the crude protein extracted from tissues of wild‐type exhibits strong PK activity; however, the mutant presented reduced protein activity. OsPK2 (PKpα1) and three other putative rice plastidic isozymes, PKpα2, PKpβ1 and PKpβ2, can interact to form heteromer. Moreover, the mutation leads to multiple metabolic disorders. Altogether, these results denote new insights into the role of *OsPK2* in plant seed development, especially in starch synthesis, compound granules formation and grain filling, which would be useful for genetic improvement of high yield and rice grain quality.

## Introduction

Starch is the essential carbohydrate storage material in plants. Because of its relevance as staple crop, rice has been used to study starch formation in seeds. Starch synthesis is a complex process involving a series of biosynthetic enzymes such as ADP‐Glc pyrophosphorylase (AGPase), starch synthase (SS), starch branching enzyme (SBE) and starch debranching enzyme (DBE) (Toyosawa *et al*., 2016). Mutation of those genes changes the appearance of the endosperm and alters the characteristics of storage starch. For instance, mutations in AGPase subunits create a wrinkled endosperm and significantly reduce the starch content (Akihiro *et al*., 2005; Lee *et al*., 2007; Tuncel *et al*., 2014; Wei *et al*., 2017). Loss‐of‐function mutations of the *GBSSI* gene produce a complete lack of endosperm amylose (Sato *et al*., 2002; Tian *et al*., 2009). Knockout of *OsSSIIIa* causes a white‐core floury endosperm and changes starch granules morphology, starch structure and gelatinization temperature (Ryoo *et al*., 2007). Mutation of *BEIIb* causes a chalkiness endosperm and has great influence on the properties of starch (Tanaka *et al*., 2004). Loss‐of‐function mutations of *ISA1*, encoding a starch DBE, cause a serious defect in amylopectin structure and a sugary endosperm (Kawagoe *et al*., 2005; Kubo *et al*., 1999). Besides starch biosynthesis enzymes, other factors associated to starch formation can also affect endosperm development in rice. For example, *FLOURY ENDOSPERM4* (*FLO4*), encoding a pyruvate orthophosphate dikinase (PPDK; EC 2.7.9.1), was shown to regulate carbon flow from starch and lipid biosynthesis during seeding stage (Kang *et al*., 2005). Some other factors such as *flo2*,* flo6*,* flo7*,* OsbZIP58*,* OsBT1* (*ADP‐glucose transporter*), *RPBF* (*rice P‐box binding factor*), *OspPGM* (*plastidic phosphor‐glucomutase*) and *RSR1* (*rice starch regulator1*) have also been shown to influence starch synthesis and endosperm development in rice (Fu and Xue, 2010; Lee *et al*., 2016; Li *et al*., 2017; Peng *et al*., 2014; She *et al*., 2010; Wang *et al*., 2013; Yamamoto *et al*., 2006; Zhang *et al*., 2016). Mutants of *SUBSTANDARD STARCH GRAIN4* (*SSG4*) *and SSG6* show enlarged starch grains (SGs) in the rice endosperm (Matsushima *et al*., 2014, 2016). All these data evidence that the genetic basis of starch synthesis and grain development implies a complex regulatory network. Therefore, identification and characterization of different rice endosperm defective mutants are necessary for further understanding this process in rice.

Pyruvate kinase (PK, ATP: pyruvate 2‐O‐phosphotransferase, EC 2.7.1.40) is a key enzyme that regulates and adjusts the final step of the glycolysis pathway. It catalyses the irreversible transfer of the high‐energy phosphate group from phosphoenolpyruvate (PEP) to ADP to synthesize pyruvate and ATP (Ambasht and Kayastha, 2002; Valentini *et al*., 2000). The glycolytic pathway provides intermediates for other crucial metabolic reactions. The product pyruvate can be decarboxylated by pyruvate dehydrogenase complex (PDC) to generate acetyl‐CoA, which can be used as the initial substrate to participate in the tricarboxylic acid (TCA) cycle to generate ATP under aerobic condition or be converted into lactic acid or ethanol under hypoxia. Besides, acetyl‐CoA can be involved in fatty acid (FA) biosynthesis and the mevalonate pathway (Grodzinski *et al*., 1999). PEP, as the intermediate product in glycolysis pathway, can also be catalysed by phosphoenolpyruvate carboxylase (PEPC) to form oxaloacetic acid (OAA) which is required for amino acid synthesis (Schwender *et al*., 2004). Therefore, PK plays an important role in the cellular metabolic flux (Zhang *et al*., 2012). PKs in plant exist as cytosolic (PKc) and plastidic (PKp) isozymes (Plaxton, 1996). In most organisms, PKs are usually present as a homotetramer (Schramm *et al*., 2000), but can also be found in a monomer (Knowles *et al*., 1989) or in complexes as homodimer (Plaxton *et al*., 2002) or in different heteromeric forms (αnβn) (Andre *et al*., 2007; Plaxton, 1989). For example, PKc in castor oil seed germinating endosperm is existed as 240 kDa heterotetramers comprised by 56 and 57 kDa subunits (Hu and Plaxton, 1996). PKp purified from castor bean had been reported to exist as a 334 kDa α3β3 heterohexameric (63.5 and 54 kDa polypeptides considered as α‐subunit and β‐subunit, respectively) (Plaxton, 1991). There are 14 genes encoding putative PK isoforms in *Arabidopsis* (Initiative, 2000). At least six PKc potato genes were identified by Southern blot analyses (Oliver *et al*., 2008). Cotton has at least 19 PKc and 14 PKp genes (Zhang and Liu, 2016). In rice, at least 11 genes have been predicted to encode putative PK isozymes (Zhang *et al*., 2012). Thus, the component forms of PK in higher plant display abundant diversity.

In plants, a ‘bottom‐up’ allosteric regulatory mechanism regulates the glycolysis pathway where PEP is considered as a potent inhibitor for ATP‐ and PPi‐dependent phosphofructokinase (Plaxton and Podestá, 2006). Meanwhile, PK as an allosteric enzyme can be inhibited by glutamate and activated by aspartate (Hu and Plaxton, 1996; Smith *et al*., 2000). Allosteric activation by 6‐p‐gluconate (G6P) in *Brassica napus* PKp has been described (Andre *et al*., 2007). The major role of PKc is to produce a precursor and ATP for various synthetic metabolic pathways (Smith *et al*., 2000). Transgenic tobacco plants lacking the leaf PKc display decreased root elongation and delayed bud differentiation and flowering (Knowles *et al*., 1998). The cotton PKc gene *GhPK6* is primarily expressed in cotton fibres, and its expression negatively correlates with the rate of fibre cell elongation (Zhang and Liu, 2016). Identification of the rice *OsPK1* gene suggested that PKc might affect plant morphological development, causing dwarfism and panicle enclosure in rice (Zhang *et al*., 2012). On the other hand, PKp is thought to provide pyruvate and ATP to support biosynthetic pathways in plastids (Andre and Benning, 2007). Besides the defects in oil accumulation and delayed germination found in *Arabidopsis pkp1* mutants (Andre *et al*., 2007; Baud *et al*., 2007), research on PKp is limited and defined roles in growth and development are unknown.

In this study, we identified a white‐core endosperm and defective grain filling mutant, *ospk2*. Map‐based cloning showed that *OsPK2* encodes a plastidic PK enzyme that can interact with three other putative PKp subunits to form a polymer which affects starch synthesis, compound granule formation and grain weight during rice seed development. This deep dissection of OsPK2 will enhance our understanding of the genetic basis of endosperm starch synthesis and grain filling in rice and provides useful information for genetic improvement of high yield and grain quality.

## Results

### The rice *ospk2* mutant displays defects in endosperm appearance and grain filling

**Figure 1:**
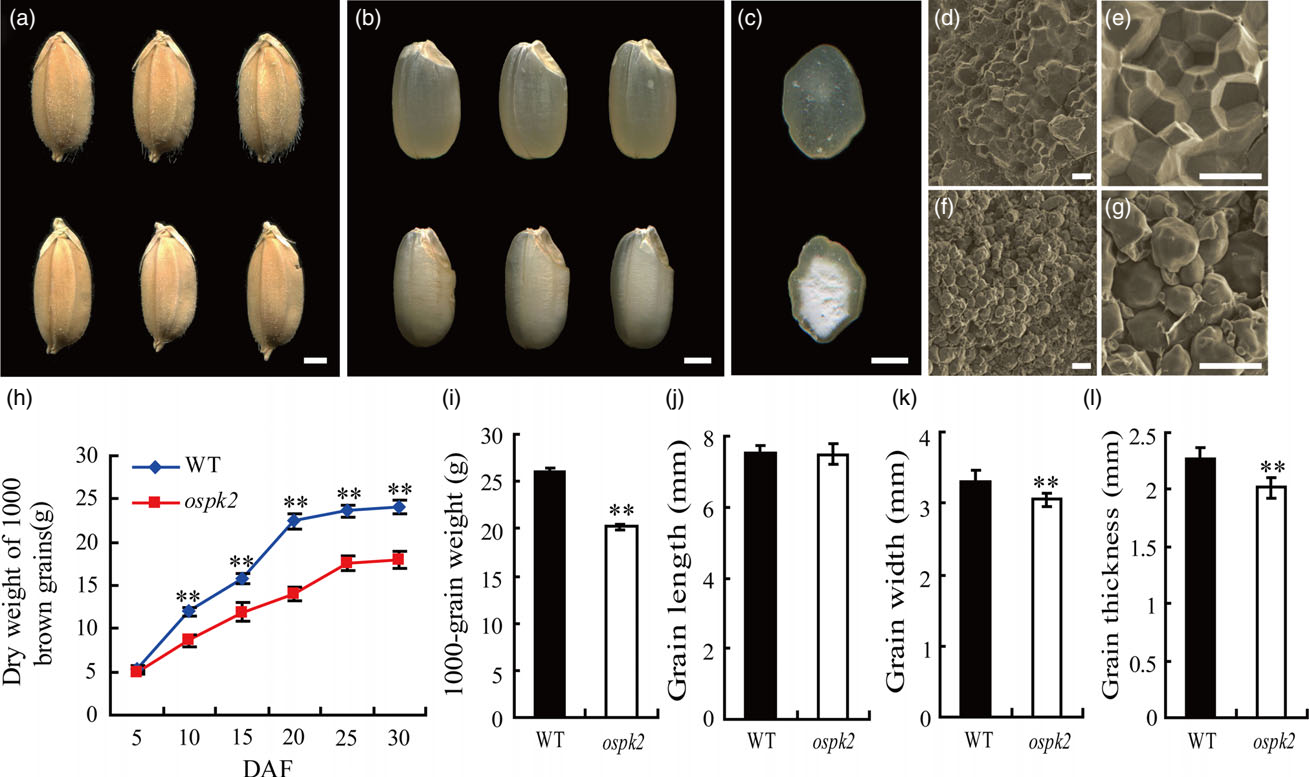
Phenotype of the *ospk2* mutant. (a–b) Appearance comparison of seeds (a) and brown rice (b) of wild‐type (WT) (above) and *ospk2* (below). (c) Transverse sections of WT (above) and *ospk2* (below) brown rice. (d–g) Scanning electron microscopy images of transverse sections of the WT (d, e) and *ospk2* mutant (f, g) grains. Scale bars, 1 mm in (a–c); 20 μm in (d, f) and 50 μm in (e, g). (h) Weight of dry grains of WT and *ospk2* at various stages of grain filling. DAF, days after fertilization. (i–l) Weight of 1000‐grains (i), grain length (j), grain width (k) and grain thickness (l) of WT and *ospk2*. Data in (h–l) are means ± SD from three biological replicates, and each replication in (i) and (h, j–l) not less than 200 and 50 seeds, respectively. Asterisks in (i–l) indicate statistical significance between the WT and the mutant, as determined by a Student's *t*‐test (**P <* *0.01).

A chalky endosperm and defective grain filling mutant, named *ospk2*, were identified from an ethyl methane sulphonate (EMS)‐mutagenized rice population. Compared to the wild‐type, the mature grains of *ospk2* displayed an opaque endosperm and presented a white‐core in the interior whereas the peripheral endosperm was transparent (Figure 1a–c). Scanning electron microscopy (SEM) analysis of the endosperm transverse sections showed that wild‐type endosperm cells were filled with densely packed and irregularly polyhedral SGs (Figure 1d,e), whereas the *ospk2* endosperm cells presented spherical and loosely packed SGs (Figure 1f,g). The grain filling rate in the *ospk2* mutant was significantly slower than the wild‐type from 10 days after fertilization (DAF), resulting in a significantly reduced grain weight and grain yield per plant in *ospk2* (Figures 1h,i and S1a). Quantification of seed size showed no significant difference in grain length but the grain width and the grain thickness of *ospk2* were reduced compared with the wild‐type (Figure 1j–l). Furthermore, germination rates of fresh harvested seeds were not significantly different between wild‐type and *ospk2*, but the buds of *ospk2* were weaker. However, the seed germination rates after 1 year of storage were strongly reduced in *ospk2* compared with wild‐type (Figure S2). Besides these grain endosperm defects, no other obvious differences in plant architecture between wild‐type and the *ospk2* mutant were observed (Figure S1b–g).

### The structure of starch compound granules is abnormal in *ospk2* endosperm cells

**Figure 2:**
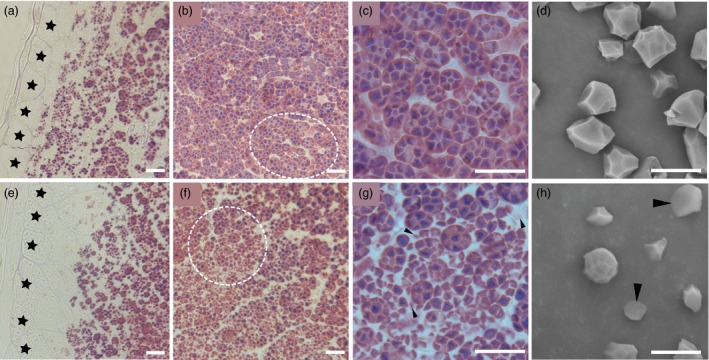
Starch granules formation in endosperm cells. (a–c) Semithin sections of wild‐type endosperm at 10 days after fertilization (DAF). (d) SEM analysis of starch or insoluble glucan granules purified from mature kernels of the wild‐type. (e–g) Semithin sections of *ospk2* mutant endosperm at 10 DAF. (h) SEM analysis of starch or insoluble glucan granules purified from mature kernels of *ospk2*. (a, e) represent the peripheral region of endosperm of wild‐type and *ospk2* mutant, respectively; (b, c) represent the central region of endosperm of wild‐type; (f, g) represent the central region of endosperm *ospk2* mutant; (c, g) are enlarged sections of the white circle region from (b) and (f), respectively. Stars in (a, e) indicate the aleurone layer cells. Arrowheads in (g) indicate smaller, weak staining or abnormal starch granules in cytosol of *ospk2*. Arrowheads in (h) represent smaller or irregular shapes starch granules in *ospk2*. Scale bars: 10 μm in (a–h).

**Figure 3:**
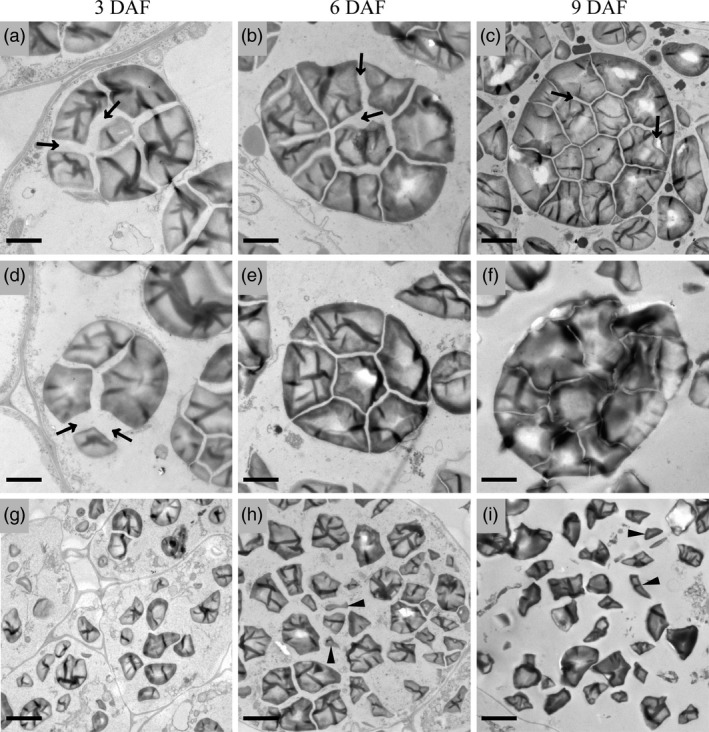
Electron micrographs depicting amyloplast development in endosperm cells of wild‐type (a–c) and *ospk2* (d–i). (a, d, g) display an amyloplast in endosperm cells of wild‐type and *ospk2* at 3 days after fertilization (DAF). (b, e, h) Amyloplast in endosperm cells of wild‐type and *ospk2* at 6 DAF. (c, f, i) Amyloplast in endosperm cells of wild‐type and *ospk2* at 9 DAF. (d–f) Represent few well‐developed amyloplasts in the endosperm cells of *ospk2* mutant. (g–i) Represent the majority phenomenon of starch granules in *ospk2* endosperm cells. Arrowheads in (a–d) show the stroma inside the amyloplast. Arrowheads in (h, i) indicate smaller, irregular shape, abnormal granules in *ospk2*. Bars: 2 μm (a–f), 5 μm (g–i).

Semithin sections of developing endosperms at 10 DAF were prepared to observe the structure of starch compound granules in wild‐type and *ospk2*. Fewer well‐developed compound granules and some immature starch granules were observed in the peripheral endosperm cells of both wild‐type and *ospk2* (Figure 2a,e). In central endosperm cells of wild‐type seeds, several polyhedral and sharp‐edged starch granules congregate to form well‐developed amyloplasts (Figure 2a,c). In central endosperm cells of *ospk2*, however, abundant single, smaller, scattered and weakly stained starch granules were also observed (Figure 2f,g). The morphology of individual starch granules was also analysed using SEM. The starch granules in wild‐type endosperm appear polygonal with sharp edges and similar size, whereas in the mutant, they were smaller and with irregular shape (Figure 2d,h). Amyloplasts at earlier developmental stages (3, 6 and 9 DAF) were observed by transmission electron microscopy. Wild‐type amyloplasts were filled with polyhedral starch granules and the interior space was quickly occupied during grain development, eventually forming a typical complex structure (Figure 3a–c). Similar to wild‐type, there were few well‐developed amyloplasts in the endosperm cells of the *ospk2* mutant (Figure 3d–f), however, the majority of the starch granules in *ospk2* endosperm cells existed as small, single and disperse granules rather than aggregated (Figure 3g–i). This was consistent with our observations of the semithin sections (Figure 2f,g). These results suggest that *OsPK2* affects the formation of starch compound granules in endosperm cells during grain development.

### Starch physicochemical properties in *ospk2*

**Figure 4:**
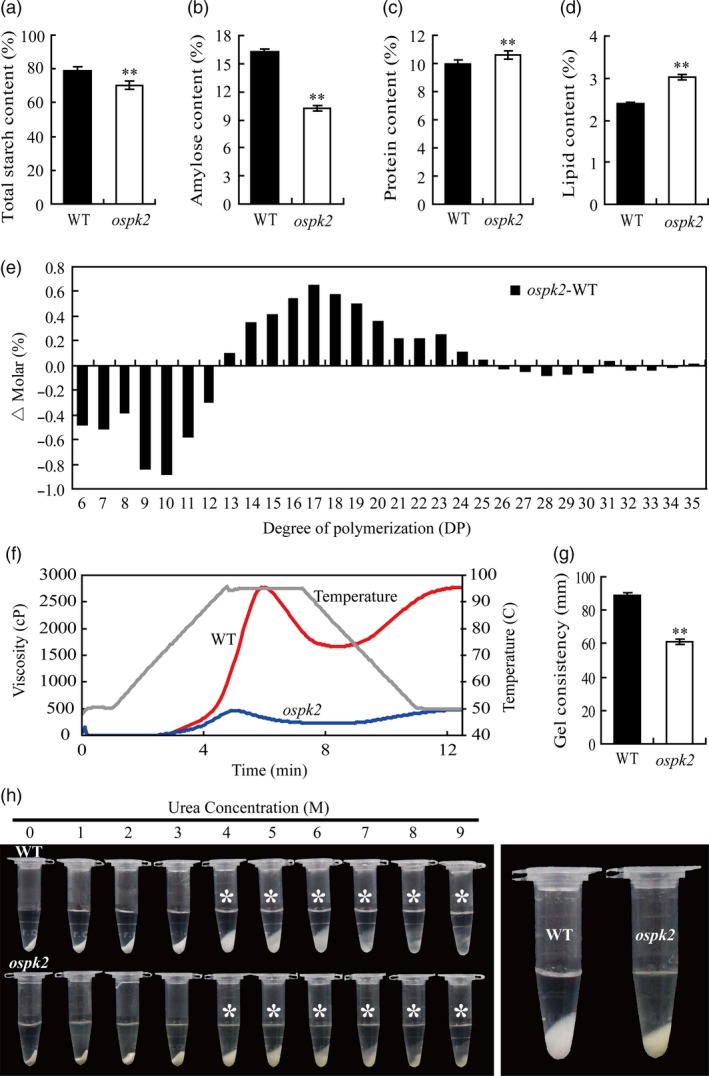
Grain characteristics and starch physicochemical characteristics in the *ospk2* mutant. (a–d) The per cent contents of total starch, amylose, protein and lipid in endosperm of wild‐type (WT) and *ospk2*. (e) Differences in the amylopectin chain length distributions between the WT and *ospk2*. (f) Pasting properties of endosperm starch of WT (red line) and *ospk2* mutant (blue line). The viscosity value at each temperature is the average of three replicates. The grey line indicates the temperature changes during the measurements. (g) The gel consistency of endosperm starch of WT and *ospk2* mutant. (h) Gelatinization characteristics of starch in urea solutions. Starch powder of WT and *ospk2* was mixed with varying concentrations (1–9 m) of urea solution. Asterisks indicate the starch of *ospk2* endosperm is more difficult to gelatinize in 4–9 m urea solution than that of WT. The most significant difference was observed for 4 m urea (right panel). Values in (a–d, g) are means ± SD from three biological replicates. Asterisks indicate statistical significance between WT and mutant, as determined by a Student's *t*‐test (*P <* *0.05; **P <* *0.01).

Because the starch granules were abnormal in both mature and developing endosperm in the *ospk2* mutant, the starch physicochemical properties were also examined. The total starch and amylose contents significantly decreased in the endosperm of *ospk*2 compared with wild‐type (Figure 4a,b). Nonetheless, the contents of protein and lipid in *ospk*2 were higher than these in wild‐type (Figure 4c,d). In the case of lipids, the contents of the C16:0 (palmitic acid), C18:0 (stearic acid), C18:1 (oleic acid), C18:2 (linoleic acid) and C22:0 (behenic acid) all increased (Figure S3). To further analyse the fine structure of amylopectin, its chain length distribution was determined. Compared to wild‐type, the proportion of short chains with degree of polymerization (DP) values between 6 and 12 decreased, whereas the proportion of intermediate chains with DP values between 13 and 25 was elevated in the *ospk2* mutant (Figure 4e).

The pasting properties of starch were analysed with a Rapid Visco Analyzer (RVA; Figure 4f). The RVA profile showed significant differences in pasting properties of the starch derived from wild‐type and *ospk2*. The viscosity of pasting starch in *ospk2* was maintained at a very low level and did not show the distinct characteristics of the peak viscosity and breakdown when the temperature rose. When the temperature was lowered, the viscosity of pasting starch in wild‐type augmented quickly and showed a high final value, whereas that of *ospk2* almost did not increase and the final viscosity was only 16.68% of that of wild‐type. The gelatinization properties of the SGs also were examined, and the result showed that SGs in *ospk2* had a significantly shorter gel consistency than wild‐type (Figure 4g). Starch solubility in urea solutions was measured. Mixing powdered starch with varying concentrations (0–9 m) of urea solutions showed that the *ospk2* starch was more difficult to gelatinize and exhibited significant different gelatinization characteristics in 4–9 m urea compared to that of wild‐type (Figure 4h). In brief, the physicochemical properties of the starch in *ospk2* endosperm were significantly different from those in wild‐type.

### Map‐based cloning and complementation of the *ospk2* mutation

**Figure 5:**
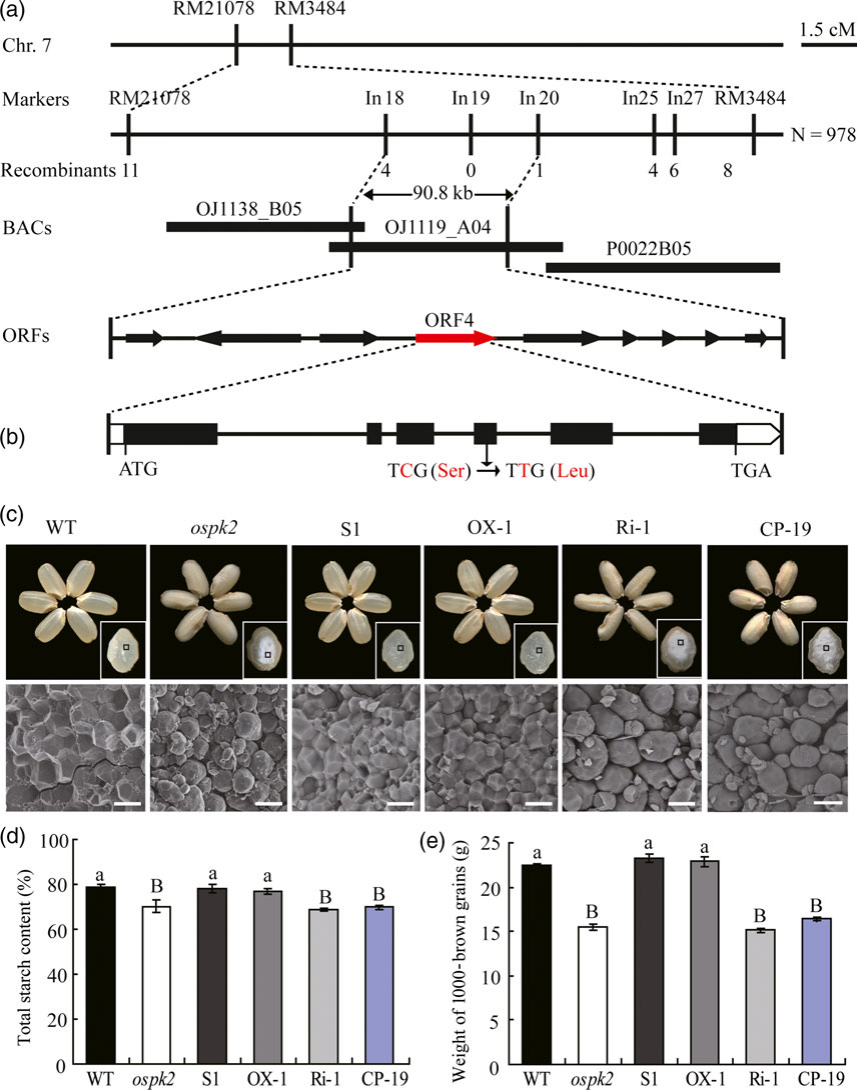
Map‐based cloning and complementation of the *ospk2* mutant. (a) Fine mapping of the *OsPK2* locus. The *OsPK2* locus was mapped to a 90.8 kb region by markers In18 and In20 on chromosome 7 (Chr.7) which contained nine predicted genes. The molecular markers and the number of recombinants are shown. cM, centimorgan; ORF, open reading frame. (b) Gene structure and mutation site in *OsPK2*. Base pair change (C to T) detected in *ospk2* in the 4th exon of Os07g0181000 causing a Ser‐296 to Leu‐296 change. ATG and TGA represent the start and stop codons, respectively. (c) Complementation of the *ospk2* mutation in transgenic lines (S1) and overexpression of *OsPK2* in *ospk2* (OX‐1) showing the restored wild‐type seed appearance and normal starch granules in endosperm, whereas RNAi (Ri‐1) and CRISPR/Cas9 mediated editing (CP‐19) of *OsPK2* in ZH11 background produce abnormal seed appearance and seeds became chalky. Bars: 20 μm. (d, e) Total starch content and 1000‐brown grains weight of grains in complementation (S1), overexpression (OX‐1), RNAi (Ri‐1) and CRISPR/Cas9 mediated editing lines (CP‐19). Data are shown as means ± SD from three biological replicates, each replication in (e) is not less than 200 seeds. Significant difference analysed by multiple‐comparison. Different letters indicate statistically significant difference (small letter, *P *<* *0.05 and capital letter, *P *<* *0.01).

To identify the gene controlling the *ospk2* phenotypes, map‐based cloning was conducted using an F_2_ mapping population that was generated by crossing the *ospk2* mutant (*japonica*) with Nanjing11 (NJ‐11, *indica*). The mutation locus was first mapped on the short arm of chromosome 7 between the simple sequence repeat (SSR) markers RM21078 and RM3484. Over 978 individuals showing the *o*s*pk2* phenotypes were chosen from this F_2_ population. The *o*s*pk2* locus was further narrowed down to a 90.8‐kb region between InDel markers In18 and In20 located on the BAC clone OSJ1119_A04, which included nine putative open reading frames (ORFs, Figure 5a; Table S1). Sequence analysis of the ORFs revealed a single nucleotide substitution from a cytosine (C) to thymine (T) on the 4th exon in ORF4 (Os07g0181000) in the *ospk2* mutant, which led a Serine^296^ replaced by a Leucine^296^ (Figure 5b). Os07g0181000 was predicted to encode a putative pyruvate kinase (PK) of about 63.58 kDa with 578 amino acids (http://smart.embl-heidelberg.de). Among a total of eleven *Oryza sativa* PK isozymes, OsPK2 (Os07g0181000, also named PKpα1) belongs to the α‐subunit type within the plastidic clade (Figure S4). Protein sequence alignment showed that the mutation site lies in a highly conserved Serine (S) residue on the PK domain among the OsPK2 homologues and that OsPK2 shares 74.33% identity with *Arabidopsis* AtPKp1 (Figure S5) which was reported as a plastidic PK involved in embryo development and control of carbon flux in maturing *Arabidopsis* seeds (Andre *et al*., 2007).

To verify whether *OsPK2* (Os07g0181000) is the gene responsible for the mutant phenotypes, a vector carrying the *OsPK2* genomic sequence including its native promoter and another vector with the *OsPK2* coding region driven by the *UBIQUTIN1* promoter were constructed and introduced into the *ospk2* mutant. The expression of *OsPK2* in complementation and overexpression transgenic lines was significantly higher than that in *ospk2* and wild‐type plants (Figure S6). Seeds harvested from independent complementation and overexpression positive transgenic T_1_ lines showed similar phenotypes to wild‐type (Figure 5c). The total starch content, 1000‐brown grain weight, grain length, width and thickness of brown rice of the complementation and overexpression lines achieved the same levels of those of wild‐type (Figures 5d,e and S7a–d). SGs in endosperm cells of these positive transgenic lines also presented the polyhedral and densely packed granule morphology, similar to those of wild‐type (Figure 5c). RNAi knock‐down plants (Ri) of *OsPK2* (Os07g0181000) were generated in a wild‐type genetic background resulting in lines with significantly lower expression levels of *OsPK2* than in wild‐type (Figure S6). The grains of those Ri lines displayed opaque endosperm (Figure 5c). The total starch content and grain weight, grain length, width and thickness of brown rice of Ri were all markedly decreased compared with wild‐type (Figures 5d,e and S7e–h). CRISPR/Cas9 technology was also applied to confirm that the phenotypic was due to the knockout of the *OsPK2* gene (Figures 5c–e and S8). Therefore, it was deduced that the abnormal grain phenotypes and starch physicochemical properties observed in the mutant were due to the mutation detected in the *OsPK2* gene.

### Expression pattern, subcellular localization and enzyme assay of *OsPK2*

**Figure 6:**
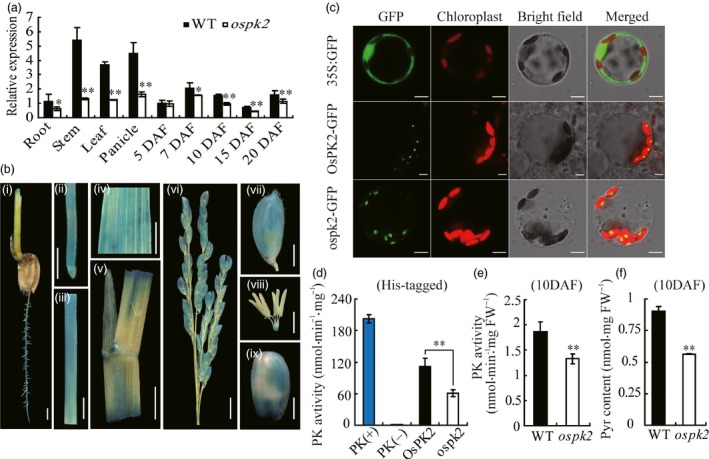
Expression pattern, subcellular localization and enzyme assay of OsPK2. (a) *OsPK2* expression level in various tissues and in developing endosperms of the WT and *ospk2*. RNA was isolated from root, stem, leaf and panicle at the heading stage and developing endosperms at 5, 7, 10, 15 and 20 days after fertilization (DAF). Values are means ± SD from three biological replicates. Asterisks indicate statistical significance between wild‐type and mutant, as determined by a Student's *t*‐test (*P < 0.05, **P <* *0.01). (b) GUS staining in root (i, ii), stem (iii), leaf (iv), leaf sheath (v), panicle (vi), glume (vii), spikelet (viii) and brown rice (ix) driven by the *OsPK2* promoter. Bar: 2 mm (i, vii–ix); 1 cm (ii–v,); 5 mm (vi). (c) Subcellular localization of OsPK2 in rice protoplast cells. From the top panel to the bottom panel: free GFP used as control; OsPK2 full‐length coding region and GFP fusion protein (OsPK2‐GFP) and ospk2 full‐length coding region and GFP fusion protein (ospk2‐GFP). Forty‐eight hours after transformation, protoplast cells were observed using a confocal laser scanning microscope. GFP signals, chlorophyll autofluorescence, bright‐field images and the merged images of GFP and chlorophyll signals are shown in each panel. Bars: 5 μm. (d) Enzyme activity assay of OsPK2 and ospk2 protein expressed in baculovirus system and purified by His‐tag. PK activity was determined by the decreasing of NADH (measured the absorbance value at 340 nm for 2 min). (e) PK activity of fresh seeds (10 DAF) from wild‐type and *ospk2*. (f) Pyruvate content of seeds (10 DAF) from wild‐type and *ospk2*. Data in (d–f) are shown as mean ± SD from three biological replicates. Asterisks indicate statistical significance as determined by a Student's *t*‐test (*P <* *0.05, **P <* *0.01).

Temporal and spatial expression analysis showed that *OsPK2* is constitutively expressed in all tested organs. Transcript levels of *OsPK2* were higher in stems, panicles and leaves but less abundant in roots and developing grains. Compared to the wild‐type, *OsPK2* transcription level in the mutant was much lower in most organs (Figure 6a). To confirm these results, a vector with the GUS reporter gene driven by the *OsPK2* promoter was transformed into rice. Histochemical analysis of GUS activity in transgenic plants corroborated that *OsPK2* presents a constitutive expression pattern (Figure 6b).

OsPK2 was predicted as a chloroplast‐targeted protein by the online tools Plant‐PLoc (http://www.csbio.sjtu.edu.cn/cgi-bin/PlantPLoc.cgi) and WoLF PSORT (http://wolfpsort.org). To verify the predicted subcellular localization and the length and position of the chloroplast‐targeting signal in OsPK2, GFP fusions driven by the CaMV 35S promoter, OsPK2‐GFP, ospk2‐GFP, OsPK2^1–451aa^‐GFP, OsPK2^1–97aa^‐GFP and OsPK2^98–578aa^‐GFP were constructed and transiently expressed in rice protoplasts and tobacco leaves. The GFP control protein was widely present in the cytoplasm and nuclei; however, both OsPK2‐GFP and ospk2‐GFP fusion proteins were localized in chloroplasts, evidenced by the exclusive colocalization with the chlorophyll autofluorescence signal (Figures 6c and S9a,b). GFP signals of OsPK2^1–451aa^‐GFP and OsPK2^1–97aa^‐GFP fusion proteins were also targeted to the chloroplasts, while the signal of OsPK2^98–578aa^‐GFP was not (Figure S9c–e). These results suggest that OsPK2 is a chloroplast‐located protein and that the 97‐amino acid N‐terminal sequence is essential for targeting OsPK2 to chloroplasts.

The proteins encoded by *OsPK2* and *ospk2* were expressed in baculovirus system and purified by His‐tag (Figure S10) and used to examine PK activity. The result showed that both OsPK2 and ospk2 present PK activity. However, the activity of ospk2 (~60.98 nmol/min/mg) was significantly lower than that of wild‐type (~112.09 nmol/min/mg) (Figure 6d). The PK activity was also detected in seeds (10 DAF) and leaves of wild‐type and *ospk2*, and the results indicated that the PK activity was also significantly decreased in the mutant (Figures 6e and S11a). Additionally, the content of pyruvate (product of PK) in seeds (10 DAF) and leaves was also significantly reduced in *ospk2* compared with wild‐type (Figures 6f and S11b). All these results demonstrate that the protein encoded by *OsPK2* has PK activity and a single amino acid substitution (Ser^296^ to Leu^296^) in the PK domain compromised its enzymatic activity.

### OsPK2 can interact with other plastidic pyruvate kinases to form heteropolymers

**Figure 7:**
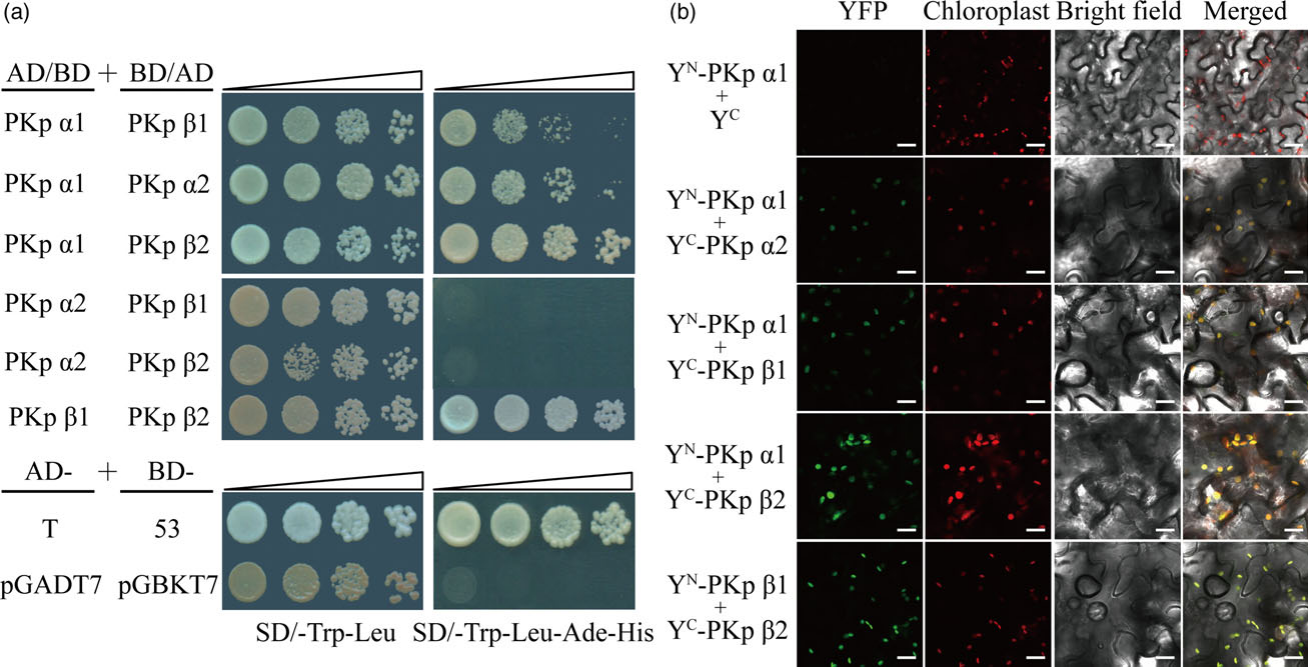
The interactions between OsPK2 and other PKps (a) yeast two‐hybrid assays showed interactions between OsPK2 (PKpα1, α1) and three putative pyruvate kinase plastidic isozymes, PKpα2 (α2), PKpβ1 (β1) and PKpβ2 (β2). Serial dilutions (10‐fold) of yeast cells expressing the indicated proteins were plated onto nonselective medium (SD/‐Leu/‐Trp) (left) or selective medium (SD/‐Leu/‐Trp/‐Ade/‐His) (right). The interactions between pGADT7‐T (T) and pGBKT7‐53 (53), pGADT7 (AD) and pGBKT7 (BD) were used as the positive and negative controls, respectively. (b) BiFC assay showed that OsPK2 (PKpα1, α1) can interact with PKpα2 (α2), PKpβ1 (β1) and PKpβ2 (β2), and PKpβ1 (β1) also can interact with PKpβ2 (β2) in the chloroplast of tobacco cells. Bars = 30 μm.

There are at least eleven genes predicted to encode putative PK isoforms in rice (Zhang *et al*., 2012), among them, *OsPK2* (*PKp*α*1*), Os03g0672300 (*PKp*α*2*, 53.48% amino acid sequence identity with OsPK2), Os01g0660300 (*PKp*β*1*, 39.47% identity with OsPK2) and Os10g0571200 (*PKp*β*2*, 39.43% identity with OsPK2) exist as plastidic PK isozymes (Baud *et al*., 2007). Transcription levels of the PK family were analysed in wild‐type and *ospk2* mutant. The results showed that the expression of *OsPK2* and *PKp*β*2* was significantly decreased, and that of *PKp*α*2*,* PKp*β*1* and two plant cytoplasmic PK isozyme genes, Os01g0276700 and Os11g0216000, were significantly increased in the *ospk2* mutant (Figure S12). Usually, PKs exist as homotetramers, but are also present as monomers, homodimers, heterodimers, heterotetramers or heteropolymers (Andre *et al*., 2007; Knowles *et al*., 1989; Plaxton, 1989; Plaxton *et al*., 1990, 2002). Approaches to test recombinant subunits of rice PKps were applied in our study. The possible interactions of PKp subunits with OsPK2 were examined by yeast two‐hybrid analysis (Y2H) and bimolecular fluorescence complementation (BiFC). Results showed that three putative PKp subunits (PKpα2, PKpβ1 and PKpβ2) can interact with OsPK2 in yeast cells (Figure 7a). The BiFC assay in tobacco epidermal cells also confirmed that OsPK2 (PKpα1) can physically interact with PKpα2, PKpβ1 and PKpβ2 (Figure 7b). In addition, Y2H and BiFC revealed that PKpβ1 and PKpβ2 can interact with each other to form a heteromer (Figure 7a,b). Moreover, we found that OsPK2 (PKpα1) and PKpα2 can interact with themselves. However, pKpβ1 and PKpβ2 cannot interact with themselves and no interaction was detected between PKpα2 and pKpβ1 or PKpβ2 (Figures 7a,b and S13). These results suggest that OsPK2 can form polymeric protein complexes *in vivo* by interacting with other PKp.

### Expression of glycolysis‐, pyruvate metabolism‐, FA metabolism‐ and starch synthesis‐related genes was affected in the *ospk2* mutant

**Figure 8:**
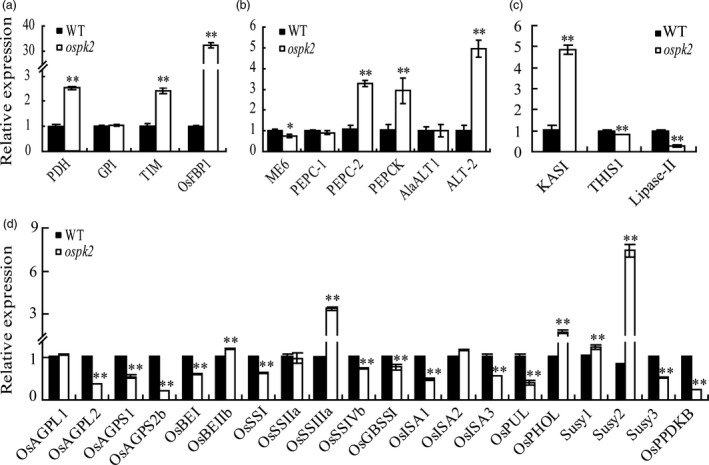
Expression analyses of genes related to glycolysis/gluconeogenesis, pyruvate/phosphoenolpyruvate metabolism, fatty acid metabolism and starch synthesis. (a) Enzyme genes of glycolysis/gluconeogenesis metabolism. *PDH* (Os04g0119400), a putative pyruvate dehydrogenase; *GPI* (Os06g0256500), glucose‐6‐phosphate isomerase; *TIM* (Os09g0535000), similar to the chloroplast precursor of triosephosphate isomerase and Os*FBP1* (Os01g0866400), a putative fructose‐1, 6‐bisphosphatase. (b) Genes related to pyruvate/PEP metabolism. *ME6* (Os01g0188400) encodes a putative chloroplastic NADP‐dependent malic enzyme; *PEPC‐1* (Os02g0244700) and *PEPC‐2* (Os01g0208700), putative phosphoenolpyruvate (PEP) carboxylases; *PEPCK* (Os10g0204400), a putative PEP carboxykinase; *OsAlaAT1* (Os10g0390500) and *ALT‐2* (Os07g0108300) encode alanine transaminases. (c) Genes involved in fatty acid synthesis and degradation. *OsKASI* (Os06g0196600) encodes a β‐ketoacyl‐[acyl carrier protein] synthase;. *THIS1* (Os01g0751600) and *Lipase‐II* (Os07g0668700) as lipolytic enzymes. (d) Genes associated with starch metabolism, including AGP genes (*OsAGPL1*,* OsAGPL2*,* OsAGPS1* and *OsAGPS2b*), starch branching enzyme genes (*OsBEI*,* OsBEIIb*), amylopectin synthesis genes (*OsSSI*,* OsSSIIa*,* OsSSIIIa* and *OsSSIVb*), amylose starch synthase gene (*OsGBSSI*), starch debranching enzyme genes (*ISA1*,*ISA2*,*ISA3*,*PUL*), sucrose synthase genes (*Susy1*,* Susy2* and *Susy3*), *OsPHOL* and *OsPPDKB*. In (a–d), RNA was isolated from wild‐type (WT) and *ospk2* grains of 12 days after fertilization. Expression levels are represented as relative to the corresponding genes in WT (set as reference value of 1) and data are shown as means ± SD from three biological replicates. Asterisks indicate statistical significance between the WT and the mutant, as determined by a Student's *t*‐test (*P <* *0.05; **P <* *0.01).

Pyruvate kinase is a key enzyme involved in the glycolytic pathway, thus, the expression of ten putative enzyme genes related to glycolysis, pyruvate metabolism and FA metabolism was analysed in developing grains of wild‐type and *ospk2*. Compared to wild‐type, expression levels of *OsFBP1* encoding fructose‐1,6‐bisphosphatase (FBPase, EC: 3.1.3.11), a key enzyme in gluconeogenesis or Calvin cycle converting fructose‐1,6‐bisphosphate to fructose‐6‐phosphate (F6P), and triose phosphate isomerase (TIM, EC: 5.3.1.1), which reversibly interconvert dihydroxyacetone phosphate (DHAP) and 3‐phosphoglyceraldehyde (GA3P), were significantly increased in the *ospk2* mutant. The expression level of a gene encoding the putative plastidic pyruvate dehydrogenase (PDH, EC: 4.1.1.1, the dehydrogenase E1 component of PDC) which converts pyruvate to acetyl‐CoA was also significantly increased. Whereas no differences were observed for the expression of the gene encoding glucose‐6‐phosphate isomerase (GPI, EC: 5.3.1.9) that interconvert G6P and F6P (Figure 8a). Otherwise, expression levels of pyruvate metabolism‐related genes *PEPC‐2* encoding PEP carboxylase, *PEPCK* for PEP carboxykinase and *ATL‐2* for alanine transaminase were all significantly increased in *ospk2*. Meanwhile, expression of the genes that encode a chloroplastic NADP‐dependent malic enzyme (ME6, Os01g0188400) and *OsAlaATL1* (Os10g0390500) was lower in the *ospk2* mutant (Figure 8b). The expression of *OsKASI*, encoding a β‐ketoacyl‐[acyl carrier protein] synthase I which participates in FA synthesis, was significantly higher in the mutant. Genes for lipolytic enzymes such as *THIS1* and *Lipase‐II* displayed lower expression in *ospk2* (Figure 8c). The expression of starch synthesis‐related genes was also evaluated in 12 DAF grains. Results showed that transcript levels of three AGP genes (*OsAGPL2*,* OsAGPS1* and *OsAGPS2b*), six amylopectin synthesis‐related genes (*OsSSI*,* OsSSIVb*,* OsGBSSI*,* OsISA1*,* OsISA3 and OsPUL*), *Susy3* and pyruvate phosphate dikinase gene *PPDKB* were all significantly lower in *ospk2* than in the wild‐type. Conversely, the transcript levels of *OsBEIIb*,* OsSSIIIa*,* OsPHOL*,* OsISA2*,* Susy1* and *Susy2* were markedly higher in *ospk2* mutant (Figure 8d).

## Discussion

### OsPK2 can interact with other plastidic PKs to form heteropolymers

Pyruvate kinase isoforms vary in different tissues and subcellular components (Andre *et al*., 2007). In cytosol, PKc exists as homotetramer in both germinating cotyledons and developing endosperm of castor oil seeds (Podestá and Plaxton, 1994; Turner *et al*., 2005). In plastids, native enzyme PKp in developing endosperm of castor oil seeds was present as a heterohexamer (α2β2; Plaxton *et al*., 1990). PKp in *Arabidopsis* was as appeared to be a 460‐kDa heterooctamer (α4β4) containing four α subunits (59.6 kDa) and four β subunits (56.8 kDa; Andre *et al*., 2007). In this work, we identified and characterized *OsPK2* encoding a PKp isozyme in rice. Subcellular localization analysis confirmed that the green fluorescent signals of OsPK2‐GFP were small dot‐like structures and only colocalized with the autofluorescent signals of chlorophyll (Figures 6c and S9), implying that OsPK2 localizes in the chloroplast nucleoids, similar to what was observed for AtPKp1 and AtPKp2 in *Arabidopsis* (Baud *et al*., 2007). In developing castor oil seeds, the N‐terminal 44 and 60 amino acids of PKp α‐ and β‐subunit contain their respective transit peptides which were responsible for their import to the leucoplast (Negm *et al*., 1995). Here, we found that the first 97 N‐terminal amino acids were necessary for the chloroplast location of OsPK2 (Figure S9). Y2H and BiFC assays indicated that OsPK2 (PKpα1) can interact with three other putative PKp subunits (PKpα2, PKpβ1 and PKpβ2) and that OsPK2 (PKpα1) and PKpα2 can also interact with themselves (Figures 7a,b and S13). These results suggest that the PKp in rice can exist as heteromeric complex. Meanwhile, BiFC assays also showed the green fluorescent signals of PKpα1&PKpα2, PKpα1&PKpβ1, PKpα1&PKpβ2 and PKpβ1&PKpβ2 overlapping with the chlorophyll autofluorescence signal in tobacco cells (Figure 7b), supporting the idea that PKpα2, PKpβ1 and PKpβ2 with OsPK2 (PKpα1) are all plastidic isozymes, consistent with the phylogenetic analysis of OsPK2 homologous proteins (Figure S4). Therefore, the enzyme complexes formed by OsPK2 subunit coordinated with three other subunits would fulfil PKp function in rice. Together, these results suggest that the PKp complex in rice might be more multifaceted and diverse than in other plants due to the multiple interactions between different subunits.

### Mutation in *OsPK2* leads to multiple disorders in metabolic processes

Pyruvate kinase catalyses the final step in glycolysis which produces ATP and NADH by breakdown glucose into pyruvate. Pyruvate can then be converted to acetyl‐CoA and NADH by the PDC which contains two distinct forms, mitochondrial (mtPDC) and plastidial (plPDC). In the cytosol, pyruvate produced by PKc is transported into mitochondria to undergo oxidative decarboxylation by mtPDC and offer carbon (acetyl‐CoA) to the TCA cycle (Smith *et al*., 2000). In plastids, the main role of PKp is to generate ATP and substrate (pyruvate) which can be catalysed by plPDC to synthesize acetyl‐CoA for FA biosynthesis (Andre *et al*., 2007). Pyruvate and acetyl‐CoA are also important products in the conversion of lipids to sugars via gluconeogenesis and the glyoxylate cycle. In addition, the intermediate PEP can be catalysed by PEPC to produce OAA which can enter the mitochondria to fuel the TCA pathway or amino acids synthesis. Malate produced by OAA in the cytosol can also enter the mitochondria (Plaxton and Leary, 2012; Sweetlove *et al*., 2010). Therefore, pyruvate is an important intermediate for sucrose/starch, lipid and amino acid metabolism. In our study, mutation in *OsPK2* significantly reduced the PK activity in *ospk2* (Figures 6e and S10b), which led to alteration of the expression of genes involved in multiple metabolic processes. For instance, the expression of *TIM*,* OsFBP1* and *PDH* was significantly higher in *ospk2* (Figure 8a). The transcription level of *OsKASI* encoding a FA synthesis enzyme was significantly higher in the mutant than in the wild‐type, while that of *THIS1* and *Lipase‐II* gene encoding the lipolytic enzyme was lower in *ospk2* (Figure 8c). In addition, expression of *Susy1* and *Susy2* was significantly higher in *ospk2* (Figure 8d). These data imply that *OsPK2* can directly or indirectly affect the TCA cycle, FA and sugar metabolism. In addition, the expression of starch synthesis‐related genes was also changed in *ospk2* (Figure 8d). Therefore, mutation in *OsPK2* leads to a variety of metabolic disorders in the *ospk2* mutant.

### *OsPK2* affects normal starch/FA biosynthesis and seed germination rate in rice

The *de novo* synthesis of FA in plants occurs predominantly in the plastid, which requires acetyl‐CoA as substrate (Harwood, 1988). Import of metabolites from the cytosol to synthesize acetyl‐CoA is needed as acetyl‐CoA is not directly imported into plastids (Roughan *et al*., 1979; Weaire and Kekwick, 1975). In plastids, pyruvate can be converted to acetyl‐CoA and NADH by the plPDC (Baud *et al*., 2007; Harwood, 1988). In our study, expression of a *PDH* gene encoding the dehydrogenase E1 component of PDC was higher in *ospk2* than in wild‐type (Figure 8a), which may lead to increased content of acetyl‐CoA and eventually increase FA content in the mutant (Figure S3). Synthesis of FA and starch in plastids requires abundant ATP. In fact, increased FA content will consume more ATP and the reduced PK activity in *ospk2* would produce less ATP, resulting in less available ATP for starch synthesis. Meanwhile, expression levels of several genes involved in starch synthesis (such as *AGPL2*,* AGPS1*,* AGPS2b* and *GBSSI*) also were reduced in the mutant (Figure 8d). All these alterations may cause the lower starch content observed in *ospk2* (Figure 4a,b). In addition, we also found that the germination rate of *ospk2* seeds after 1‐year storage declined largely (Figure S2). This might be caused by the increased amount of FA in *ospk2* (Figure S3). The lower germination rate was consistent with the results described for the mutation of *AtPKp1* in *Arabidopsis* (Baud *et al*., 2007). Together, these results denote new insights into the role of *OsPK2* in plant seed development, especially in starch/FA biosynthesis, compound granules formation and grain filling, and provide useful grounds for potential genetic improvement of high yield and grain quality in rice.

## Materials and methods

### Plant materials and growth conditions

The *ospk2* mutant was created from *O. sativa* subsp. *japonica* cv. Zhonghua 11 (ZH11) by EMS mutagenesis. The F_2_ population for gene mapping was generated by crossing the *ospk2* mutant with the *indica* rice cv. Nanjing11 (NJ11). Plants were grown under natural conditions at China National Rice Research Institute experimental fields in Fuyang, Hangzhou, China.

### Map‐based cloning of the *OsPK2* gene

To map the *OsPK2* locus, white‐core endosperm seeds were selected from the F_2_ population produced from the cross between the *ospk2* and NJ‐11. More than 170 polymorphic SSR markers evenly distributed over the whole genome were selected to identify markers linked to *OsPK2*. Further molecular markers were developed based on nucleotide polymorphisms between wild‐type and NJ‐11 in the corresponding regions (Table S2). To fine map the *OsPK2* locus, 978 recessive individuals with the mutant phenotype were selected from the F2 population.

### Microscopy analysis

The brown rice of wild‐type and the *ospk2* mutant was cut transversely, and the ruptured transverse surface was coated with gold to prepare samples. Images were obtained with a HITACHI S‐3400N scanning electron microscope (Hitachi, Tokyo, Japan; http://www.hitachi‐ hitec.com). To examine the development of compound granules, transverse sections (approximately 1 mm in thickness) of wild‐type and *ospk2* endosperms at 10 DAF were used to prepare semithin sections (800 nm). Samples were stained with I_2_‐KI for 5 s and subsequently examined under a light microscope (Nikon Eclipse 80i, Japan; http://www.nikon.com). All treatments of samples were followed as described previously (Peng *et al*., 2014). To observe the ultrastructure of amyloplasts, developing seeds (3–12 DAF) from wild‐type and *ospk2* were analysed by transmission electron microscope (H‐7650; Hitachi, http://www.hitachi.com). Samples were treated as described by Takemoto *et al*. (2002).

### Physicochemical properties of the starch in rice grains

The total starch content of the rice powder was measured using a starch assay kit (Megazyme, Wicklow, Ireland; http://www.megazyme.com). Amylose content was determined following the method depicted by Liu *et al*. (2009). Lipid and protein contents in the grains were measured according to the method described by Kang *et al*. (2005). Content of FA was determined by gas chromatography‐mass spectrometer (GC‐MS, Li *et al*., 2006). To determine the starch pasting properties, 3 g of milled rice power (0.5 mm or less, 14% moisture basis) was transferred into a container with 25 mL of distilled water. The sample was mixed and measured with a Rapid Visco Analyzer (RVA Techmaster; Newport Scientific, Narrabeen, Australia), applying the protocol described by the manufacturer. To determine the chain length distribution of amylopectin, 5 mg of rice flour was digested with *Pseudomonas amyloderamosa* isoamylase (Megazyme), then analysed using the capillary electrophoresis (PA800 plus pharmaceutical analysis system; Beckman Coulter, http://www.beckmancoulter.com). The swelling and gelatinization properties of endosperm starch in urea solution were measured according to the method described by Nishi *et al*. (2001). All parameters related to physicochemical properties included three biological replications.

### RNA extraction and real‐time PCR analysis

Total RNA was extracted from different plant tissues (root, stem, leaf, panicle and seeds at 5, 7, 10, 15, 20 DAF) using the Trizol reagent (Life Technologies). A 2‐μg portion of total RNA was reverse‐transcribed into cDNA according to the manufacturer's instructions (ReverTra Ace qPCR RT Kit; Toyobo). qRT–PCR was performed using the SYBR Green Real‐time PCR Master Mix (Toyobo). PCR programme was as follows: 95 °C for 30 s, 40 cycles of 95 °C for 5 s, 60 °C for 35 s and 95 °C for 15 s. Gene expression was calculated by the 2^−ΔΔCT^ method using the *Ubiquitin* gene (Os03g0234200) as internal control. The primer sequences used in this analysis are listed in Table S2.

### Plasmid construction and rice transformation

For complementation of the *ospk2* mutant, the wild‐type *OsPK2* genomic fragment from ZH11, including its native promoter, was amplified by PCR (all primers used for plasmid construction are listed in Table S2) and then cloned into the binary vector pCAMBIA1300 to generate the complementation vector. A vector bearing the *OsPK2* cDNA sequence driven by *UBIQUTIN1* promoter was cloned into the binary vector pCAMBIA1390 to generate the overexpression vector. To obtain the RNAi vector, a 481 bp fragment from the *OsPK2* cDNA from ZH11 was cloned into the binary vector LH‐FAD2‐1390RNAi under the control of the maize *UBIQUTIN1* promoter. The CRISPR target of OsPK2 (GGCCGACCTGAGGGAGAAC) was cloned into a guide sgRNA (U3 gRNA) by restriction site (*Bsa*I), and gateway recombination was used to incorporate gRNA into the pYLCRISPR/Cas9‐MH/B vector. These plasmids were introduced into *Agrobacterium tumiefaciens* strain EHA105. Complementation and overexpression vectors were transformed into *ospk2*, and the RNAi and the CRISPR/Cas9 vectors were transformed into wild‐type plants. The genotypes of the transgenic plants were determined by PCR amplification of the specific transgenic fragment.

### Subcellular localization of OsPK2 protein

To verify the subcellular localization of OsPK2, the coding regions without a termination codon from wild‐type and *ospk2* were amplified by PCR and cloned into the pAN580 and pCAMBIA1305‐GFP vectors to generate the OsPK2‐GFP, and ospk2‐GFP constructs. Other four vectors bearing the fragment of OsPK2^1–97aa^, OsPK2^98−578aa^ and OsPK2^1–451aa^ were also constructed into pCAMBIA1305‐GFP. Stem and leaf sheath tissues from 12‐day‐old green rice seedlings were sliced into 0.5 mm long segments to prepare protoplasts. The OsPK2‐GFP, ospk2‐GFP and pAN580 vectors were transformed into rice protoplasts and incubated in dark at 28 °C for 2 days following previous description (Chen *et al*., 2006). The other constructs were introduced into *Agrobacterium strain* GV3101 which was then infiltrated into 3‐week‐old leaf epidermal cells of *Nicotiana benthamiana* (tobacco). After infiltration for 48 h, GFP fluorescence in tobacco leaves was observed with a LSM710 confocal laser scanning microscope (Carl Zeiss AG, Oberkochen, Germany).

### GUS staining

The putative promoter region of *OsPK2* (~2‐kb upstream of ATG) was amplified by PCR and cloned into the *EcoR*I/*Nco*I sites of pCAMBIA1305. The resultant construct was transformed into ZH11 calli, and independent lines of positive T_1_ transgenic progeny were used to detect GUS activity. Tissues were submerged in GUS staining solution (10 mm EDTA, 0.1% Triton X‐100, 1 mm 5‐bromo‐4‐chloro‐3‐indoyl‐ b‐D‐glucuronide, 100 mm sodium phosphate (pH 7.0), 2.5 mm K_4_Fe(CN)_6_ and 2.5 mm K_3_Fe(CN)_6_ at 37 °C for 12–15 h). After incubation, tissues were discoloured several times in pure ethyl alcohol.

### Protein expression, enzyme assay and pyruvate content detection

To validate the PK activity of OsPK2, the full‐length CDS of *OsPK2* and *ospk2* were cloned into the Bac‐to‐Bac baculovirus expression vector PFAST‐BAC I (Thermo Fisher Scientific). Protein expression and purification by His‐tag using the baculovirus expression system followed the method depicted by King and Possee (1992). On the other hand, a PK crude enzyme solution was also extracted from fresh leaves and 10 DAF seeds of wild‐type and *ospk2* according to a previous report (Baud *et al*., 2007). First, 0.1 g of plant material was ground in liquid nitrogen and homogenized with 1 mL extraction buffer consisting of 50 mm HEPES‐KOH buffer (pH 8.0), 100 mm KCL, 5 mm MgCl_2_, 20 mm NaF, 1 mm EDTA, 0.1% (v/v) Triton X‐100, 20% (v/v) glycerol, 5% (w/v) polyethylene glycol 8000, 1 mm 2,2′‐dithiodipyridine and 1% (w/v) insoluble poly(vinylpolypyrrolidone). The homogenate was centrifuged at 14 000 ***g*** for 10 min at 4 °C, and the supernatant was used for enzyme activity. Both expressed protein and crude enzyme solution extracted from fresh tissues were used to measure PK activity by the method of coupling reaction of pyruvate and the conversion of NADH to NAD^+^. PKp reaction solution contained 100 mm Hepes‐KOH (pH 8.25), 50 mm KCl, 10 mm MgCl_2_, 5% (w/v) polyethylene glycol 8000, 1 mm dithiothreitol, 2 mm PEP, 0.30 mm NADH, 2.5 mm ADP and 2 U/mL desalted rabbit muscle lactate dehydrogenase (Sigma, L1254; Sigma‐Aldrich Co., St. Louis, MO). About 0.5 U/mL PK from rabbit muscle (Sigma, P9136; Sigma‐Aldrich Co.) was used as the positive control, and the inactivated His‐OsPK2 by heat treated at 95 °C for 20 min served as negative control. The declined absorbance values at 340 nm of the reaction solution from 20 s to 2 min 20 s were analysed with a microplate spectrophotometer (Infinite 200 PRO; TECAN, Mannedorf, Switzerland) to calculate PK activity. Simultaneously, pyruvate content in leaf and 10 DAF seeds of wild‐type and *ospk2* was determined by the 2,4‐dinitrobenzene hydrazine colorimetric method using the pyruvate assay kit (TC0750‐100T; Beijing Leagene Biotechnology Co., Ltd., http://www.leagene.com). First, 0.5 g of fresh leaves and 10 DAF seeds were ground in liquid nitrogen and homogenized with 9 mL of 8% trichloroacetic acid in ice for 30 min, then centrifuged at 4000 ***g*** for 10 min at 4 °C. About 1.5 mL of supernatant was mix well with 0.5 mL 0.1% 2,4‐dinitrobenzene hydrazine, then 2.5 mL of 2 m NaOH was added with adequate mixing. After 5 min, the absorbance value at 520 nm was recorded using a microplate spectrophotometer (Infinite200 PRO; TECAN).

### Yeast two‐hybrid assay and BiFC assay

The coding region of *OsPK2* without a termination codon was amplified by PCR and cloned into pGBKT7 and pGADT7 (Clontech, http://www.clontech.com). Coding regions of several rice PK genes were also cloned into the same vectors (Table S2). Yeast transformation and selecting procedures were accomplished according to the manufacturer's guidebook (Clontech). For BiFC assays, the full‐length cDNAs without a termination codon of Os03g0672300 (named *PKp*α*2*), Os01g0660300 (*PKp*β*1*), Os10g0571200 (*PKp*β*2*) and *OsPK2* (Os07g0181000, also named *PKp*α*1*) were cloned into the binary vectors pSPYCE or pSPYNE to create the constructors pPKα2‐YFPC, pPKβ1‐YFPC, pPKβ2‐YFPC and pOsPK2–YFPN, respectively. The plasmids were transiently expressed in tobacco leaves following the method described by Waadt and Kudla (2008). A confocal laser scanning microscope (Zeiss LSM710) was used to detect YFP fluorescent signals after 48 h post‐transfection. Primers used in this assay are listed in Table S2.

## Author contributions

All authors contributed to the design and implementation of the experimental strategy and participated in the data collection. P. H. and X. W. designed the experiments. Y. C., S. L., X. W., G. J., Z. S., Y. W., G. S., L. X., C. P. and S. T. carried out the experimental work. Y. C., S. L. and X. W. wrote the first draft of the manuscript which was critically revised and implemented by P. H., S. T. and J. X. All authors discussed the results and commented on the final version of the manuscript.

## Conflict of interest

The authors declare that they have no competing financial interest.

## Supporting information


**Figure S1** Characteristics of adult wild‐type and *ospk2* plants.
**Figure S2** The germinability of fresh harvested and 1 year storage seeds of wild‐type and *ospk2*.
**Figure S3** Fatty acid composition in mature seeds.
**Figure S4** Phylogenetic analysis of *OsPK2*.
**Figure S5** Amino acid sequence alignment between OsPK2 and other homologous proteins.
**Figure S6** qRT‐PCR analysis of *OsPK2* in transgenic plants.
**Figure S7** Analysis of *OsPK2* transgenic plants.
**Figure S8** Analysis of *OsPK2* CRISPR‐CAS9‐mediated editing.
**Figure S9** Subcellular localization of OsPK2 and truncated OsPK2 in tobacco cells.
**Figure S10** SDS‐PAGE and western‐blot analysis of his‐OsPK2 and his‐ospk2 purified from baculovirus expression system under native condition.
**Figure S11** PK activity assay in fresh leaves.
**Figure S12** qRT‐PCR analysis of genes encoding putative PK in rice.
**Figure S13** Yeast two‐hybrid assays showed that both OsPK2 (PKpα1) and PKpβ1 can interact with itself.
**Table S1** Gene products of the nine predicted ORFs in the fine mapping region.
**Table S2** Primers used in this study.
