# Supplementary material for: OsPK2 encodes a plastidic pyruvate kinase involved in rice endosperm starch synthesis, compound granule formation and grain filling
Source: Plant Biotechnol J. 2018 Apr 17;16(11):1878–91. doi: 10.1111/pbi.12923 (PMC6181219; doi:10.1111/pbi.12923)
Supplement: Supplementary file 1 — Figure S1 Characteristics of adult wild‐type and ospk2 plants. Figure S2 The germinability of fresh harvested and 1 year storage seeds of wild‐type and ospk2. Figure S3 Fatty acid composition in mature seeds. Figure S4 Phylogenetic analysis of OsPK2. Figure S5 Amino acid sequence alignment between OsPK2 and other homologous proteins. Figure S6 qRT‐PCR analysis of OsPK2 in transgenic plants. Figure S7 Analysis of OsPK2 transgenic plants. Figure S8 Analysis of OsPK2 CRISPR‐CAS9‐mediated editing. Figure S9 Subcellular localization of OsPK2 and truncated OsPK2 in tobacco cells. Figure S10 SDS‐PAGE and western‐blot analysis of his‐OsPK2 and his‐ospk2 purified from baculovirus expression system under native condition. Figure S11 PK activity assay in fresh leaves. Figure S12 qRT‐PCR analysis of genes encoding putative PK in rice. Figure S13 Yeast two‐hybrid assays showed that both OsPK2 (PKpα1) and PKpβ1 can interact with itself. Table S1 Gene products of the nine predicted ORFs in the fine mapping region. Table S2 Primers used in this study. [file PBI-16-1878-s001.pdf]

Supplementary materials:

***OsPK2*, encodes a plastidic pyruvate kinase involved in rice endosperm starch synthesis, compound granule formation and grain filling**

Yicong Cai [1, #], Sanfeng Li [1, #], Guiai Jiao [1], Zhonghua Sheng [1], Yawen Wu [1], Gaoneng Shao [1], Lihong Xie [1], Cheng Peng [2], Junfeng Xu [2], Shaoqing Tang [1], Xiangjin Wei [1, *], Peisong Hu [1, *]

[1] State Key Laboratory of Rice Biology, China National Rice Research Institute, Hangzhou, 310006, China

[2] State Key Laboratory Breeding Base for Zhejiang Sustainable Pest and Disease Control, Institute of Quality and Standard for Agro-products, Zhejiang Academy of Agricultural Sciences; Hangzhou, 310021, China

[#]These authors contributed equally to this work.

[*] **Authors for correspondence:**

Peisong Hu: peisonghu@126.com, hupeisong@caas.cn

Xiangjin Wei: weixiangjin@caas.cn

Phone: +86-571-63370221, Fax: +86-571-63371532

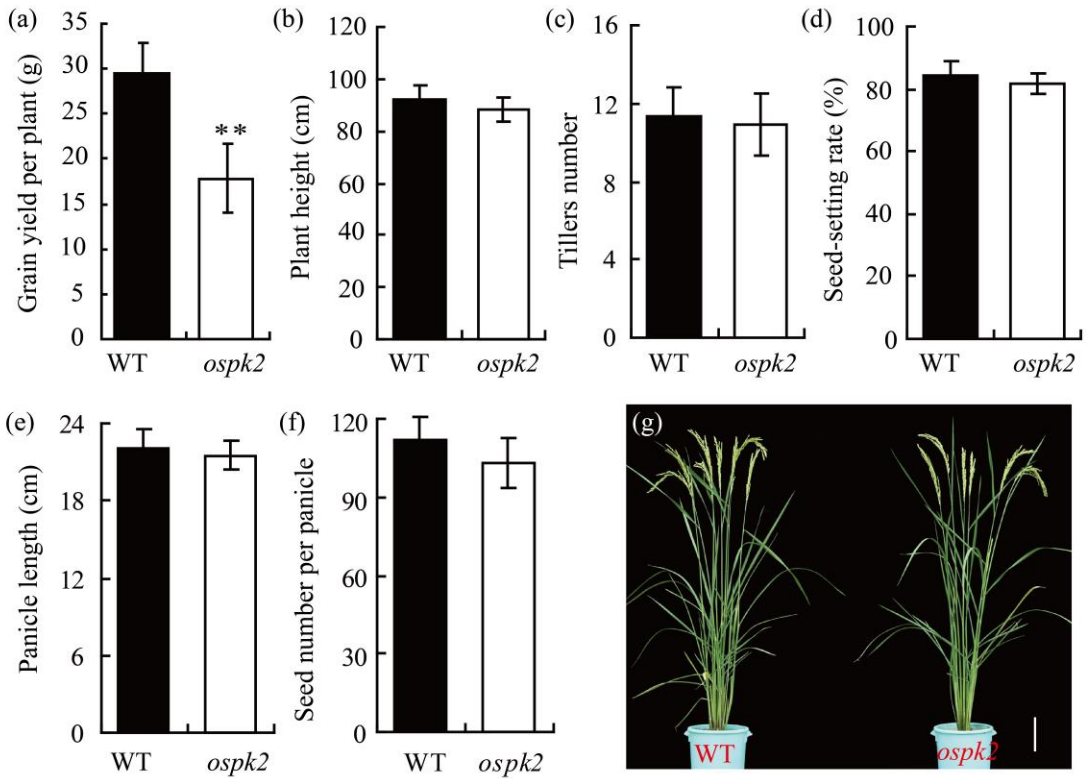

Figure S1. Characteristics of wild-type and *ospk2* plants. (a) Grain yield per plant. (b) Plant height. (c) Tillers number. (d) Seed-setting rate. (e) Panicle length. (f) Seed number per panicle. Values are means ± *SD* from three biological replicates, not less than 10 plants in each replication. The asterisks indicate statistical significance compared with the wild-type, as determined by a Student's *t*-test (**$P < 0.01$). (g) The plants of wild-type and *ospk2* mutant after heading, bar: 10cm.

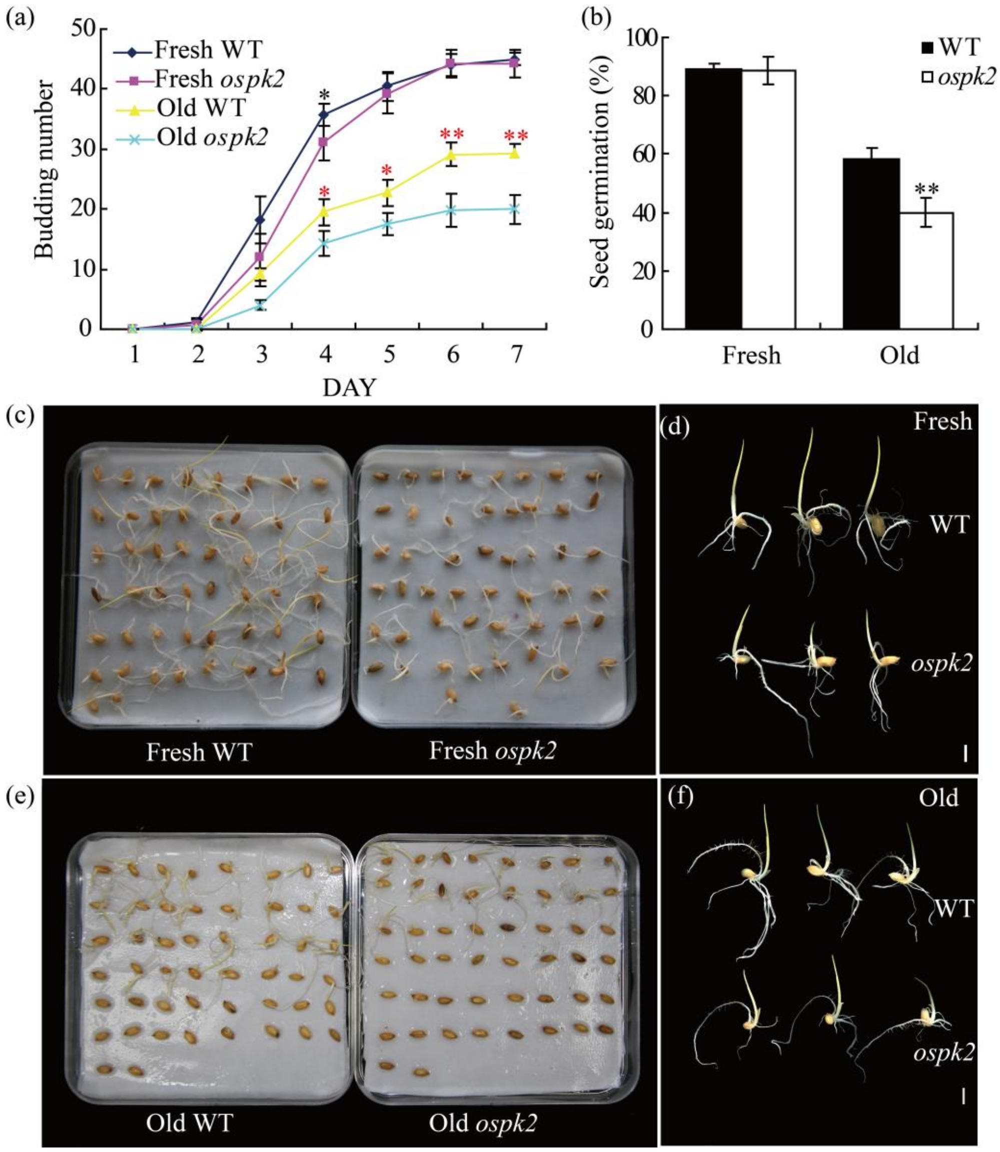

Figure S2. The germinability of fresh harvested and one year storage seeds of wild-type and *ospk2*. The experiment was carried out in dark condition with 80% humidity at 28℃. (a) Budding number of wild-type and *ospk2* at various stages of seeds germination. 'Fresh' and 'Old' represent fresh harvested and one year storage seeds, respectively. (b) Seed germinating rate of wild-type and *ospk2* after 7days induced. (c-f) The photo of fresh harvested (c, d) and one year storage (e, f) seeds after 7d germinated. Bars: 5mm (d, f). Values in (a) and (b) are means ± *SD* from three biological replicates, not less than 50 seeds in each replication. Asterisks in (a) and (b) indicate statistical significance of difference between wild-type and *ospk2*, as determined by a Student's *t*-test (* $P < 0.05$, ** $P < 0.01$).

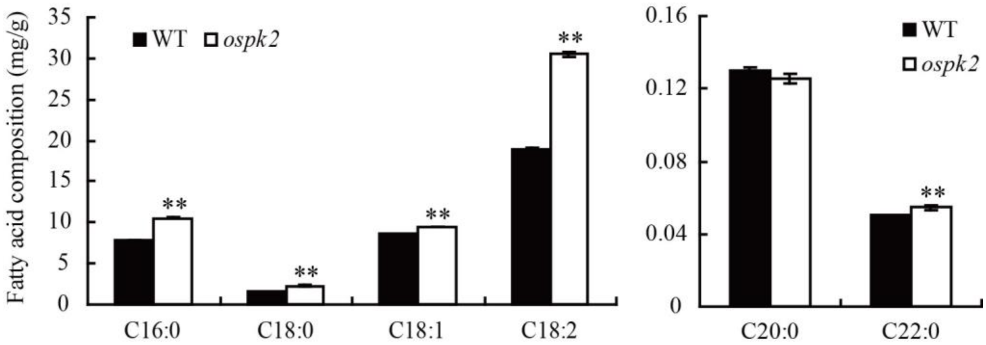

Figure S3. Fatty acid composition in mature seeds. The measurement includes long-chain (16 and 18 carbons) and very-long-chain (20 and 22 carbons) fatty acids. C16.0, C18.0, C18:1, C18:2, C20:0 and C22:0 stand for palmitic acid, stearic acid, oleic acid and linoleic acid, arachic acid and behenic acid, respectively. Data are presented as means ± *SD* from three biological replicates. Significant difference analyzed by a Student's *t*-test (***P* < 0.01).

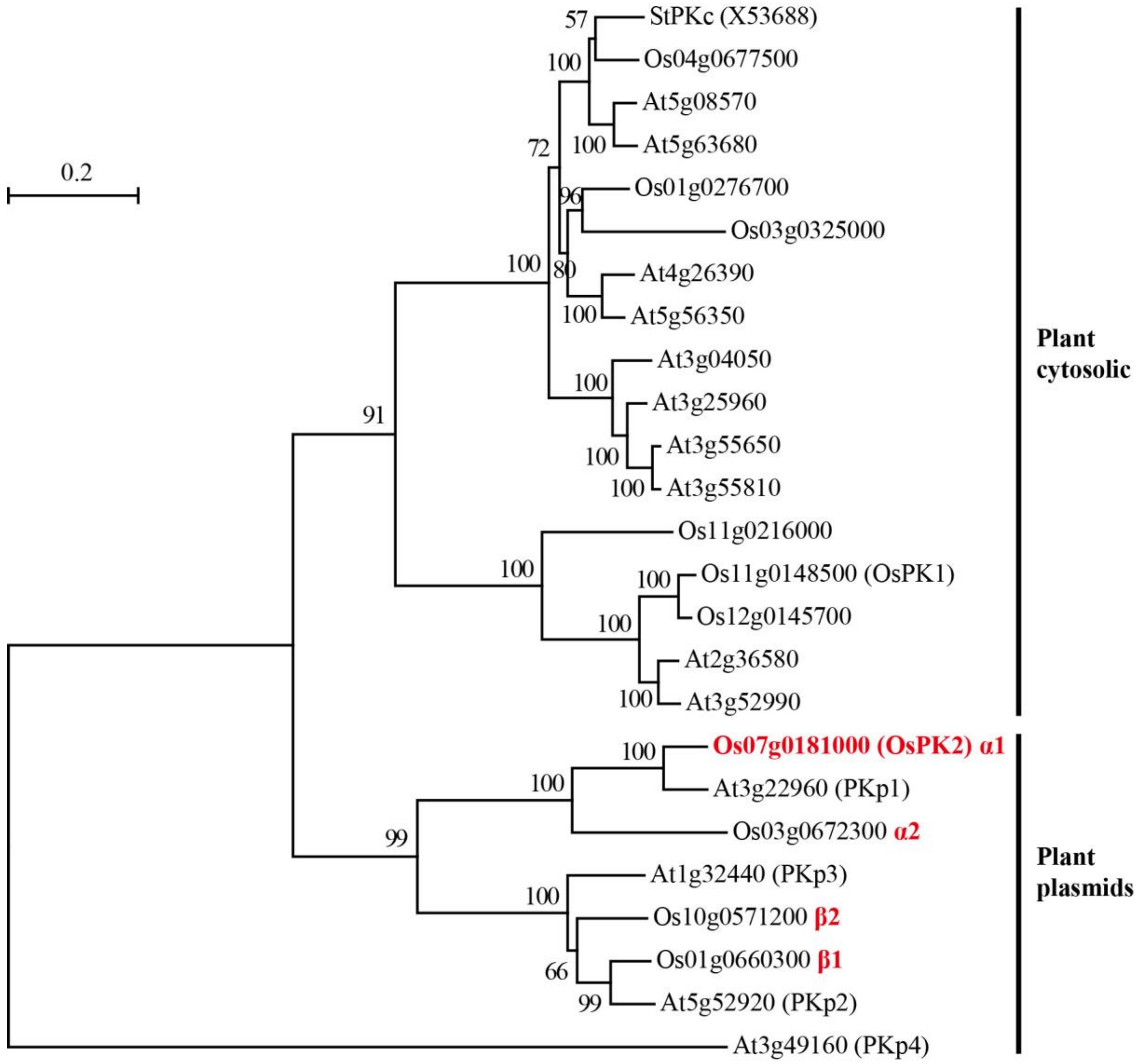

Figure S4. Phylogenetic analysis of *OsPK2*. The neighbor-joining tree was generated with MEGA5.2 after aligning the full-length protein sequences. The phylogeny indicates a distinction between the cytosolic and plastidic forms. Abbreviations are as follows: At, *Arabidopsis thaliana*; St, *Solanum tuberosum* (potato); α, subunit α; β, subunit β.

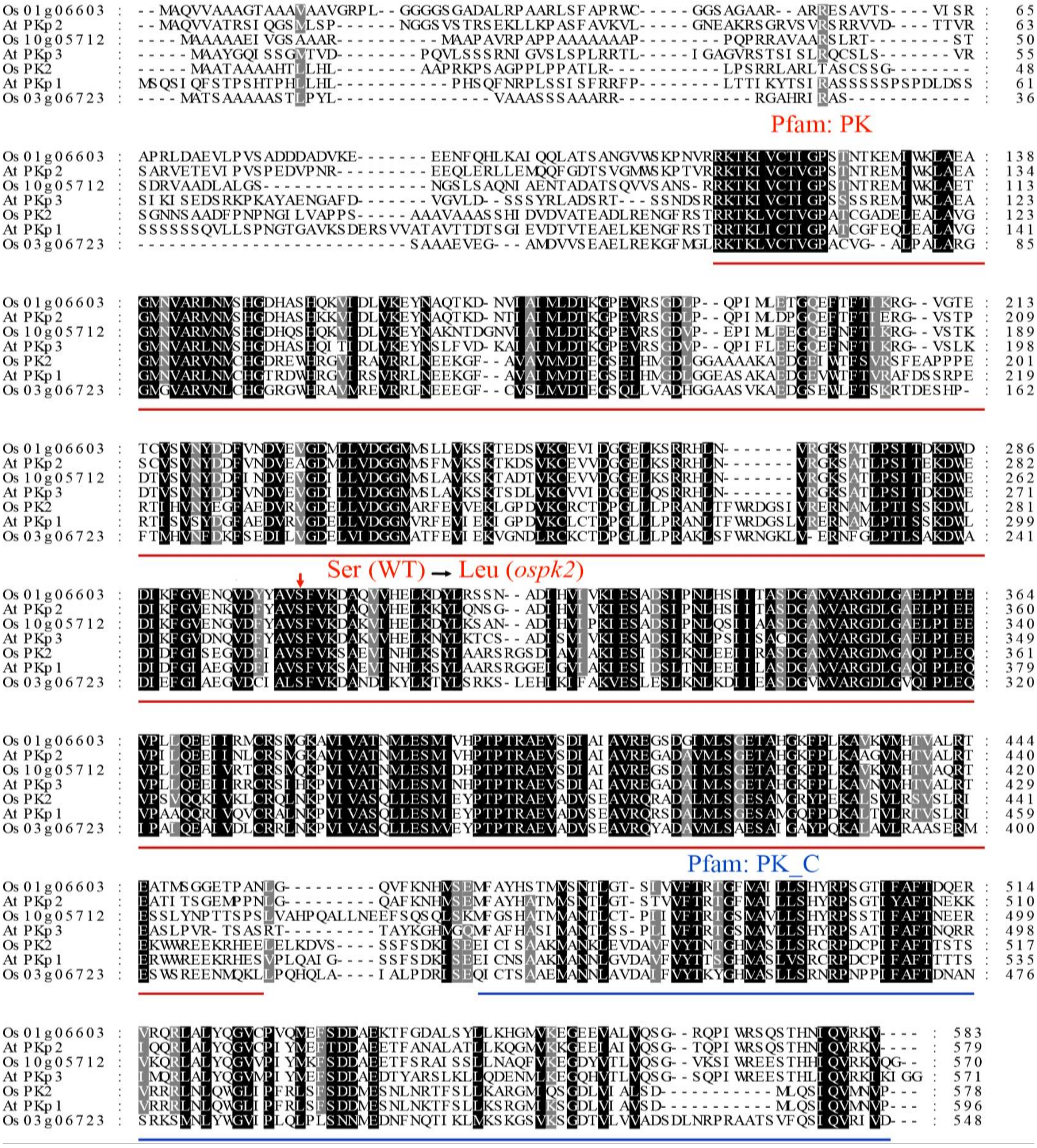

Figure S5 Amino acid sequence alignment between OsPK2 and other homologous proteins. The sequences alignment was generated with ClustalX2. White alphabet with black background means 100% identity, white alphabet with grey background represents 80% identity, and black alphabet with light grey background stands for 60% identity. OsPK2 shared 39.47%, 53.48%, 39.43%, 74.33%, 37.29%, 37.73% amino acid identity with Os01g0660300 (PKpβ1), Os03g0672300 (PKpα2), Os10g0571200 (PKpβ2), AtPKp1 (At3g22960), AtPKp2 (At5g52920) and AtPKp3 (At1g32440), respectively. Amino acid sequence underline with red color and blue

color represent the domain of PK and PK_C. The red arrowhead shows the mutation site of *ospk2*.

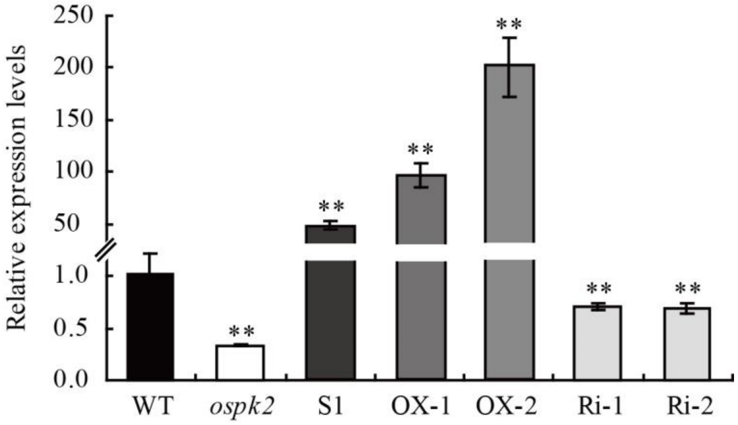

Figure S6. qRT-PCR analysis of *OsPK2* in transgenic plants. S1, the complemented transgenic line; OX-1 and OX-2, overexpression the *OsPK2* gene in the *ospk2* mutant; Ri-1 and R1-2, two *OsPK2* RNAi lines. RNA was isolated from leaf of WT, *ospk2* and transgenic plants. The value of ubiquitin mRNA was used as an internal control. Relative expression was calculated expressions of *OsPK2* in WT was set as reference value of 1. Data are presented as mean ± *SD* from three biological replicates. Asterisks indicate statistical significance compared with the wild-type, as determined by a Student's *t*-test (***P* < 0.01).

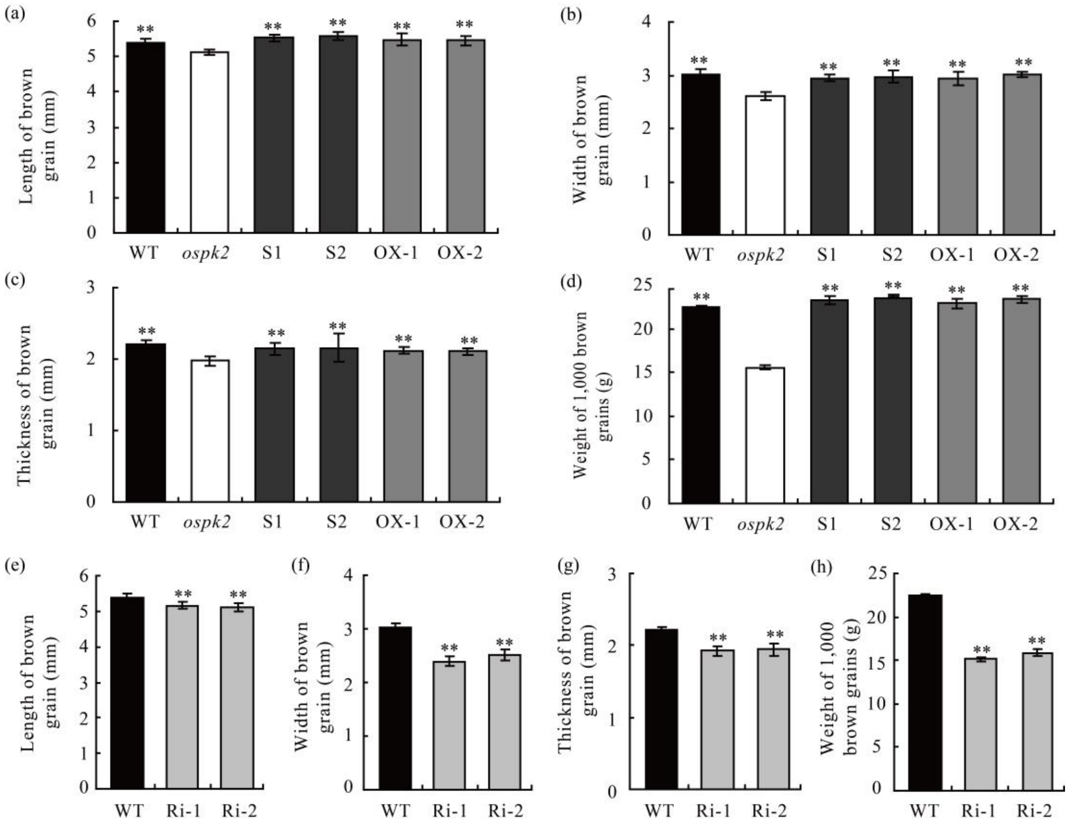

Figure S7. Analysis of *OsPK2* transgenic plants. (a-d) The grain length (a), width (b), thickness (c) and 1000-grain weight (d) of brown grains of *OsPK2* complemented and overexpression transgenic plants. (e-h) The grain length (e), width (f), thickness (g) and 1000-grain weight (h) of brown grains of *OsPK2*-RNAi transgenic plants. S1 and S2 are two complemented transgenic lines. OX-1 and OX-2 are two overexpression transgenic lines. Ri-1 and R1-2 are two *OsPK2* RNAi lines. Data are shown as mean ± *SD* from three biological replicates. Each replication was not less than 50 (a-c, e-g) and 200 (d, h) seeds, respectively. Asterisks in (a-d) indicated the statistical significance from the compare of complemented lines, OX lines and WT with *ospk2*, and asterisks in (e-f) indicated the statistical significance between Ri lines and WT, determined by a Student's *t*-test (***P* < 0.01).

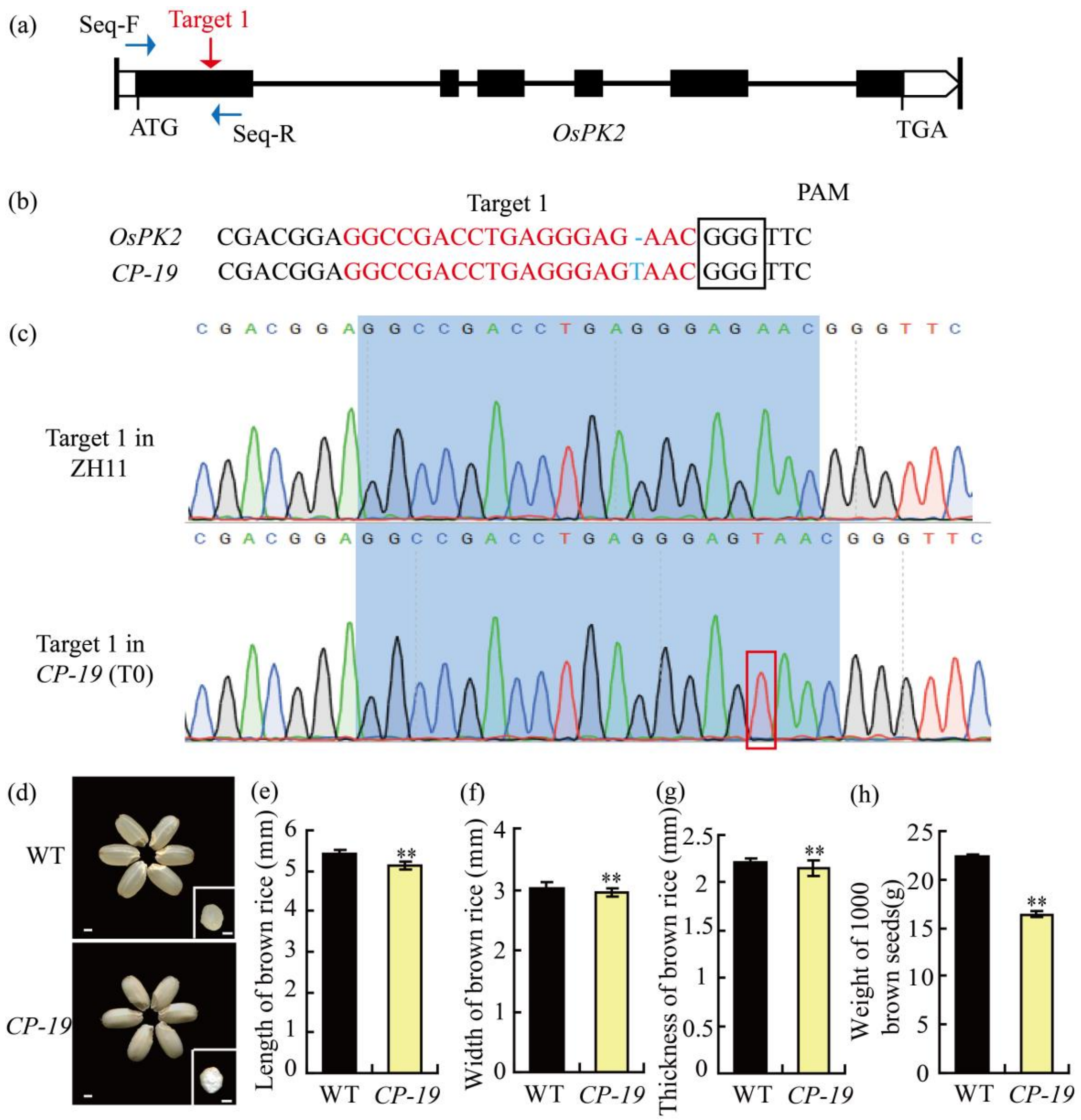

Figure S8. Analysis of *OsPK2* CRISPR/CAS9 mediated editing. (a) *OsPK2* structure. (b) PAM sequence and *OsPK2* editing. (c) Target region sequencing in ZH11 and T_0 plant. (d) Appearance and transverse sections of mature seeds of wild-type and *OsPK2* knock out line (*CP-19*). Bars: 1mm. (e-h) The grain length (e), width (f), thickness (g) and weight of 1,000 grains (h) of brown rice of wild-type and *OsPK2* knock out line (*CP-19*). *CP-19* is a homozygous mutant line of *OsPK2* by CRISPR/CAS9 mediated editing. Data in (e-h) are shown as mean ± *SD* from three biological replicates, and each replication was not less than 50 (e-g) and 200 (h) seeds, respectively. Asterisks indicate statistical significance as determined by a Student's *t*-test (***P* < 0.01).

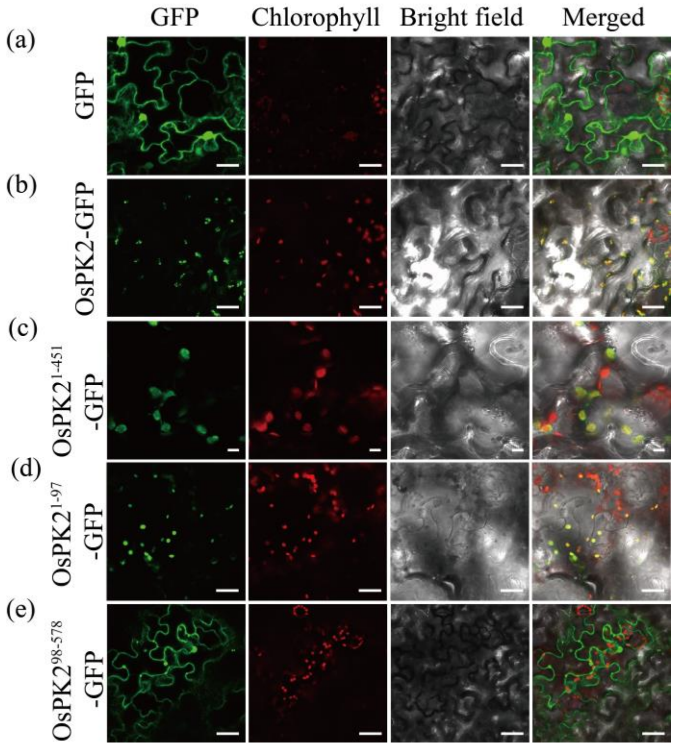

Figure S9. Subcellular localization of OsPK2 and truncated OsPK2 in tobacco cells. (a) free GFP used as a control; (b) OsPK2 full-length coding region and GFP fusion protein (OsPK2-GFP); (c) the N-terminal 451AA of OsPK2 and GFP fusion protein (OsPK2$^{1–451}$-GFP); (d) the N-terminal 97AA of OsPK2 and GFP fusion protein (OsPK2$^{1–97}$-GFP) and (e) OsPK2-GFP fusion protein lacking the N-terminal 97AA (OsPK2$^{98\text{-}578}$-GFP). 48h after transformation, tobacco cells were observed using a confocal laser scanning microscope. GFP signals, chlorophyll autofluorescence, bright-field images, and the merged images of GFP signal and chlorophyll signals are shown in each panel. Bars: 10μm.

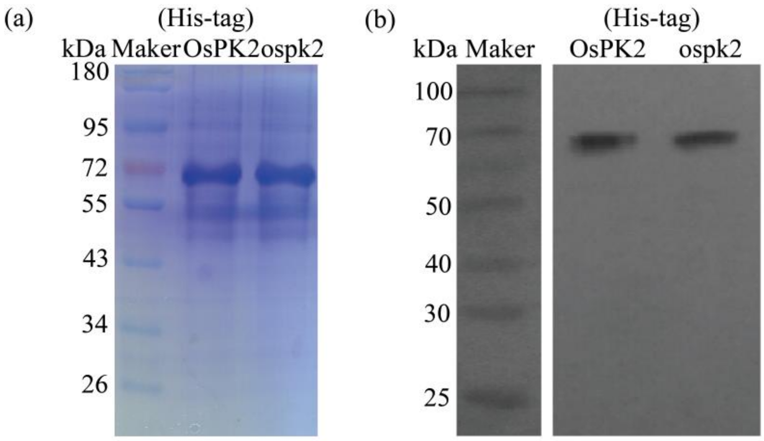

Figure S10. SDS-PAGE (a) and western-blot (b) analysis of his-OsPK2 and his-ospk2 purified from baculovirus expression system under native condition. (a) The full-length CDS of OSPK2 from wild-type and *ospk2* were cloned into the baculovirus expression vector PFAST-BAC I. Proteins with his-tag were purified from Bac-to-Bac baculovirus expression system, and subjected to SDS–PAGE with 10% (w/v) separating gel. Add 8μg protein in each lane for electrophoresis. The gel was stained with Coomassie blue R-250. The molecular masses of the band in SDS-PAGE were similar to the value of predicted size of OsPK2 (63.5kDa). (b) Western blot analysis of OsPK2 and ospk2 protein by with His-tag antibody. Samples with 8 μg/lane were electrophoresed on SDS-PACE with 10% (w/v) separating gel and blot transferred to a polyvinylidene difluoride (PVDF) membrane. The bands in membrane were closed to the molecular masse of predicted size of OsPK2.

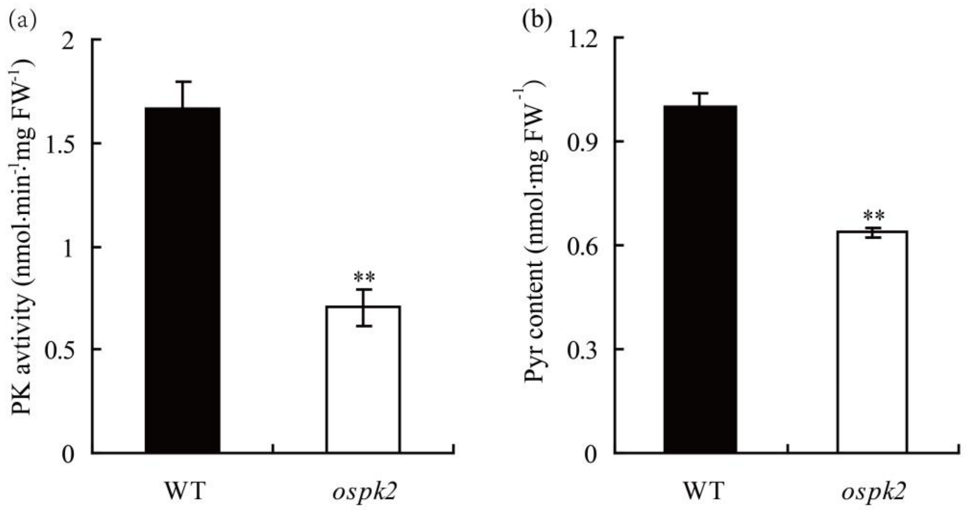

Figure S11. PK activity assay in leaves. (a) PK activity of the crude enzyme solution extracted from fresh leaves from WT and *ospk2*. (b) Pyruvate content of leaves from WT and *ospk2*. Data are shown as mean ± *SD* from three independent replicates. Asterisks indicate statistical significance as determined by a Student's *t*-test (**P < 0.01).

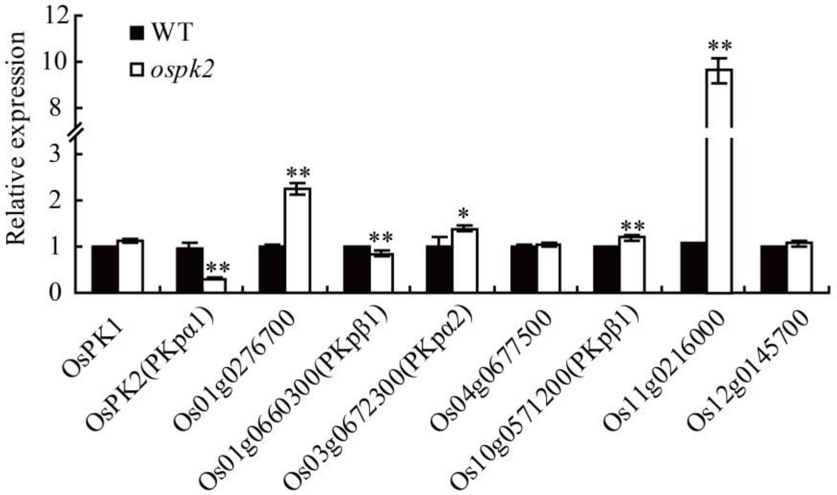

Figure S12. qRT-PCR analysis of genes encoding putative PK in rice. Data are presented as mean ± *SD* from three independent replicates. RNA was isolated from WT and *ospk2* panicle at the heading stage. Asterisks indicate statistical significance compared with the wild-type, as determined by a Student's *t*-test (**P* < 0.05, ***P* < 0.01).

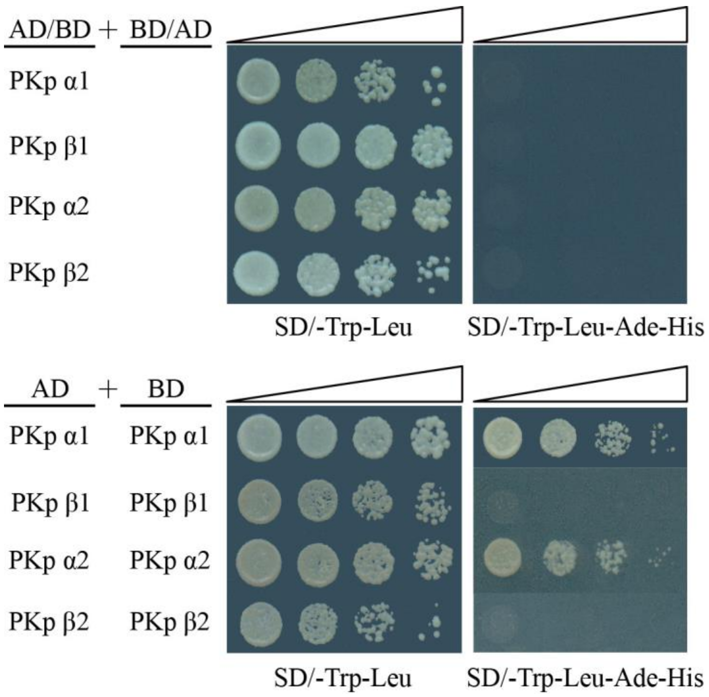

Figure S13. Yeast two-hybrid assays showed that both OsPK2 (PKpα1) and PKpβ1 can interact with itself. *OsPK2* and other PK members were fused to activation domain (AD) or binding domain (BD). Serial dilutions (10-fold) of yeast cells expressing the indicated proteins were plated onto nonselective medium (*SD*/-Leu/-Trp) (left) or selective medium (*SD*/-Leu/-Trp/-Ade/ -His) (right).

Table S1. Gene products of the nine predicted ORFs in the fine mapping region.

Number	Locus	Description of function
ORF1	Os07g08300	major facilitator superfamily antiporter
ORF2	Os07g08320	RNA recognition motif containing protein
ORF3	Os07g08330	ribosomal protein L4
ORF4	Os07g08340	pyruvate kinase
ORF5	Os07g08350	C4-dicarboxylate transporter/malic acid transport protein
ORF6	Os07g08360	expressed protein
ORF7	Os07g08370	expressed protein
ORF8	Os07g08380	expressed protein
ORF9	Os07g08390	plant-specific domain TIGR01615 family protein

Table S2. Primers used in this study.

Use	Primer name	Sequence (5’ to 3’)
**Fine mapping**	RM3484-F	TCCGGTCGTCCTCATCGTATCC
	RM3484-R	GCCCTCTTGCTCCCACATCG
	RM8010-F	CAGCTTCAGCTCCTAATGGTTGC
	RM8010-R	GCCTCTCAGAGCCTTCTTCTTCC
	RM21078-F	CAAGCTGCCGTGTTCTACTGG
	RM21078-R	GCACACAACAAGAGACAGTAACATGC
	RM7479-F	AGCGCCACATGGTGGCTTAGG
	RM7479-R	CACCGACTTATGCGAGTCGTTCG
	In18F	TTTGGTGCTGCCATTGTCG
	In18R	GCCTCGGAGCTGACGTTGA
	In19F	CGCATTGTACTACCTTCTC
	In19R	TATTCCACCAGTTTTCCTC
	In20F	AAAGGAAAAGGAGAAATAC
	In20R	AGTTTGGTGTCATGGATAT
	In25F	GCTGCTATTGCTATCCATCC
	In25R	ATCTGCCTGTGCTTGCTCT
	In27F	GCTTGGCTTTGACGAACCCTC
	In27R	CGACATCGCCGCCTACCAGA
**Sequencing**	F1	CATTGTTGTAGCTCAGTGTCTGTC
	R1	TGAGGCTCGCTCAACTTGAT
	F2	TGTTCTAGGGCTCATGGCTT
	R2	GTGAAGTGAATGAACCCGAC
	F3	TCCATCACATGAAAACTTTCCT
	R3	GGCAGCTAAGCTCACCTCCG
	F4	AGGCCGACCTGAGGGAGAA
	R4	TGCTAGATAGCGGTCAAGCC
	F5	GGCAGTCATAAGCTCTGTTC
	R5	CAGTTCAACCATCAAACCATGT
	F6	AGACTTTGGAATTTCTGAAGGC
	R6	TCGATTCAAGAAGCTGCGAC
	F7	GGAGATAATCCGTGCTTCAG
	R7	TACAACGCGCTGGAAGTTGC
	F8	GTGTTTGTTTGTTCTGCCCA
	R8	AACCATGCAGTAACTGTGCCAA
**Binary vector construction**	1300-OsPK2-KpnI-F	CTCAATTCGAGCTCGGTACCTTGATTCATTGTAGGAGGAG
	1300-OsPK2-HindIII-R	GGCCAGTGCCAAGCTTATGGCTGAGACTTGAGATTA
	1390-OsPK2-kpnI-F	GGGGTACCATGGCCGCCACCGCCGCCG
	1390-OsPK2-SpeI-R	GGACTAGTTCAAGGTACGTTCATGACC
	1390RNAi-OsPK2-KpnI-F	GGGGTACCGGAGGAGATAATCCGTGC
	1390RNAi-OsPK2-SacI-R	CGAGCTCCATGTGGCCAGTGTTTGT

	1390RNAi-OsPK2-PstI-F	AACTGCAGCATGTGGCCAGTGTTTGT
	1390RNAi-OsPK2-BamHI-R	CGGGATCCGGAGGAGATAATCCGTGC
**Crispr Cas9**	U3-OsPK2-F	GGCAGGCCGACCTGAGGGAGAAC
	U3-OsPK2-R	AAACGTTCTCCCTCAGGTCGGCC
	Cas9-check-F	TCATCAAAAGCATCCTCTC
	Cas9-check-R	AGCACGCATTCCTACTCAC
**GUS**	1305-OsPK2-EcoRI-F	CCATGATTACGAATTCCGCAGTGAGCGTTGTCTT
	1305-OsPK2-NcoI-R	CTCAGATCTACCATGGCCCTTCTCCTCGTTGAGC
**Subcellular localization**	1305GFP -OsPK2-SpeI-F	GCCCAGATCAACTAGTATGGCCGCCACCGCCGCCGC
	1305GFP -OsPK2-BamHI-R	TGCTCACCATGGATCCAGGTACGTTCATGACCTGGA
	OsPK2(1-97)-BamHI-R	TGCTCACCATGGATCCCCGGAACCCGTTCTCCCTCA
	OsPK2Δ1-97-SpeI-F	GCCCAGATCAACTAGTATGAGCACGCGGCGCACCAA
	OsPK(21-451)-BamHI-R	TGCTCACCATGGATCCATGGCGCTTCTCCTCTCTCC
	OsPK2Δ1-451-SpeI-F	GCCCAGATCAACTAGTATGGAGGAACTGGAACTTAA
**Protein expression**	OsPK2- PFAST-BACI-F	GAAGCGCGCGGAATTCATGGCCGCCACCGCCGCCG
	OsPK2- PFAST-BACI-R	CCAGGTCATGAACGTACCTCATCACCATCACCATCACT GAAAGCTTG
**Yeast two-hybrid assays**	pGBKT7-OsPk2-EcoRI-F	CATGGAGGCCGAATTCATGGCCGCCACCGCCGCCGC
	pGBKT7-OsPk2-PstI-R	TAGTTATGCGGCCGCTGCAGTCAAGGTACGTTCATGACCT
	pGADT7-OsPk2-EcoRI-F	GGAGGCCAGTGAATTCATGGCCGCCACCGCCGCCGC
	pGADT7-OsPk2-BamHI-R	CGAGCTCGATGGATCCTCAAGGTACGTTCATGACCT
	pGBKT7-PKpα2-EcoRI-F	CATGGAGGCCGAATTCATGGCAACCTCCGCCGCCG
	pGBKT7-PKpα2-PstI-R	TAGTTATGCGGCCGCTGCAGCTAGTCCACTATTCGAACT
	pGADT7-PKpα2-EcoRI-F	GGAGGCCAGTGAATTCATGGCAACCTCCGCCGCCG
	pGADT7-PKpα2-BamHI-R	CGAGCTCGATGGATCCCTAGTCCACTATTCGAACT
	pGBKT7-PKpβ1-EcoRI-F	CATGGAGGCCGAATTCATGGCGGCGGCGGCGGCTG
	pGBKT7-PKpβ1-PstI-R	TAGTTATGCGGCCGCTGCAGTTAGCCCTGGACTTTCCTC
	pGADT7-PKpβ1-EcoRI-F	GGAGGCCAGTGAATTCATGGCGGCGGCGGCGGCTG
	pGADT7-PKpβ1-BamHI-R	CGAGCTCGATGGATCCTTAGCCCTGGACTTTCCTC
	pGBKT7-PKpβ2-EcoRI-F	CATGGAGGCCGAATTCATGGCGCAGGTGGTGGCTG
	pGBKT7-PKpβ2-PstI-R	TAGTTATGCGGCCGCTGCAGTCAAACCTTCCTGACCTGA
	pGADT7-OsPk2-EcoRI-F	GGAGGCCAGTGAATTCATGGCGCAGGTGGTGGCTG
	pGADT7-PKpβ2-BamHI-R	CGAGCTCGATGGATCCTCAAACCTTCCTGACCTGA
**BiFc Assays**	pSPYNE-OsPK2-F	GCCTACTAGTGGATCCATGGCCGCCACCGCCGCCGC
	pSPYNE-OsPK2-R	GAGCGGTACCCTCGAGAGGTACGTTCATGACCTGGA
	pSPYCE-OsPK2-F	CGATAGTACTGTCGACATGGCCGCCACCGCCGCCGC
	pSPYCE-OsPK2-R	CCCGGGAGCGGTACCAGGTACGTTCATGACCTGGA
	pSPYNE-PKpα2-F	GCCTACTAGTGGATCCATGGCAACCTCCGCCGCCG
	pSPYNE-PKpα2-R	GAGCGGTACCCTCGAGCTAGTCCACTATTCGAACT
	pSPYCE-PKpα2-F	CGATAGTACTGTCGACATGGCAACCTCCGCCGCCG
	pSPYCE- PKpα2-R	CCCGGGAGCGGTACCCTAGTCCACTATTCGAACT
	pSPYNE-PKpβ1-F	GCCTACTAGTGGATCCATGGCGGCGGCGGCGGCTG
	pSPYNE-PKpβ1-R	GAGCGGTACCCTCGAGTTAGCCCTGGACTTTCCTC

	pSPYCE-PKpβ1-F	CGATAGTACTGTCGACATGGCGGCGGCGGCGGCTG
	pSPYCE- PKpβ1-R	CCCGGGAGCGGTACCTTAGCCCTGGACTTTCCTC
	pSPYNE-PKpβ2-F	GCCTACTAGTGGATCCATGGCGCAGGTGGTGGCTG
	pSPYNE-PKpβ2-R	GAGCGGTACCCTCGAGTCAAACCTTCCTGACCTGA
	pSPYCE-PKpβ2-F	CGATAGTACTGTCGACATGGCGCAGGTGGTGGCTG
	pSPYCE- PKpβ2-R	CCCGGGAGCGGTACCTCAAACCTTCCTGACCTGA
**Q-RT-PCR**	qRT-PCR-OsPK2-F	CATGAGGAACTGGAACTTAAA
	qRT-PCR-OsPK2-R	GACGAAAACGGCATCTAC
	qRT-PCR-Ubi-F	GCTCCGTGGCGGTATCAT
	qRT-PCR-Ubi-R	CGGCAGTTGACAGCCCTAG
	LOC_Os01g16960-RT-F	GGTGTCCCAAACAAGATTGA
	LOC_Os01g16960- RT-R	TCTCAACCTTTGACATCAGCA
	LOC_Os01g47080-RT-F	TGTTCGTGGAAAGAGCG
	LOC_Os01g47080-RT-R	AAAGAAACGGCATAGTAGTCA
	LOC_Os03g20880-RT-F	GACCTTATGCTGGTTCGA
	LOC_Os03g20880-RT-R	CATCAACATTAGCAACACCT
	LOC_Os03g46910-RT-F	ACAGACCCAGGTTTGCTTCT
	LOC_Os03g46910-RT-R	CCAATCCTTTGCTGACAATG
	LOC_Os04g58110-RT-F	GCTCTGTCGTTTGTCCGTAA
	LOC_Os04g58110-RT-R	TTTCAACCTTTGACATCAGCTT
	LOC_Os10g42100-RT-F	GCAGTTCGTGAAGGTTCTGA
	LOC_Os10g42100-RT-R	GGATTCTGTTCTCTGTGCCA
	LOC_Os11g05110-RT-F	AAGCCTGCTGTTGTTACTCGT
	LOC_Os11g05110-RT-R	TCACTCCCGTCAAGTACAGC
	LOC_Os11g10980-RT-F	GGGCTCTGATCCAACAAAGT
	LOC_Os11g10980-RT-R	CTCCTCAGTACTCGCCATCA
	LOC_Os12g05110-RT-F	AAGTATAGGCCAACCATGCC
	LOC_Os12g05110-RT-R	GAGCGATTGTCTTGCTTCAA
